# Purple non‐sulphur bacteria and plant production: benefits for fertilization, stress resistance and the environment

**DOI:** 10.1111/1751-7915.13474

**Published:** 2019-08-21

**Authors:** Myrsini Sakarika, Janne Spanoghe, Yixing Sui, Eva Wambacq, Oliver Grunert, Geert Haesaert, Marc Spiller, Siegfried E. Vlaeminck

**Affiliations:** ^1^ Research Group of Sustainable Air, Energy and Water Technology Department of Bioscience Engineering University of Antwerp Groenenborgerlaan 171 2020 Antwerpen Belgium; ^2^ Department of Plants and Crops Faculty of Bioscience Engineering Ghent University V. Vaerwyckweg 1 9000 Ghent Belgium; ^3^ Greenyard Horticulture Belgium NV Skaldenstraat 7a 9042 Gent Belgium

## Abstract

Purple non‐sulphur bacteria (PNSB) are phototrophic microorganisms, which increasingly gain attention in plant production due to their ability to produce and accumulate high‐value compounds that are beneficial for plant growth. Remarkable features of PNSB include the accumulation of polyphosphate, the production of pigments and vitamins and the production of plant growth‐promoting substances (PGPSs). Scattered case studies on the application of PNSB for plant cultivation have been reported for decades, yet a comprehensive overview is lacking. This review highlights the potential of using PNSB in plant production, with emphasis on three key performance indicators (KPIs): fertilization, resistance to stress (biotic and abiotic) and environmental benefits. PNSB have the potential to enhance plant growth performance, increase the yield and quality of edible plant biomass, boost the resistance to environmental stresses, bioremediate heavy metals and mitigate greenhouse gas emissions. Here, the mechanisms responsible for these attributes are discussed. A distinction is made between the use of living and dead PNSB cells, where critical interpretation of existing literature revealed the better performance of living cells. Finally, this review presents research gaps that remain yet to be elucidated and proposes a roadmap for future research and implementation paving the way for a more sustainable crop production.

## Graphical abstract

Purple non‐sulfur bacteria (PNSB) are beneficial for plant growth, due to their ability to produce and accumulate high‐value compounds. These phototrophs have the potential to enhance plant growth performance, increase the yield and quality of edible plant biomass, boost the resistance to environmental stresses, bioremediate heavy metals and mitigate greenhouse gas emissions. Therefore, the use of PNSB in agricultural applications can pave the way for a more sustainable crop production.

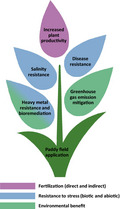

## Introduction

Agriculture is strongly challenged in the 21st century as a result of the growing world population (FAO, 2009) and the increasing demand for natural resources (Giljum *et al*., 2015). The rising need for agricultural products results not only in an increase in arable land demand and thus the deforestation of uniquely biodiverse ecosystems (such as Amazon in Brazil) but also an increased consumption of fertilizers (FAO, 2015). In order to sustainably meet the future global food demand, multiple mitigation measures are needed to decrease the impact on the environment (Pikaar *et al*., 2018). Adopting improved fertilization strategies (i.e. better‐quality fertilizers), reducing the stress to plants and environment (e.g. heavy metals) and reducing the environmental impact of crop production (e.g. greenhouse gas emissions) will lead on to meeting this goal. A conventional approach to enhance crop yield at the greatest extent is by using synthetic fertilizers, which contain all necessary nutrients in their inorganic form. When applied to the plant, these inorganic nutrients are readily accessible and thus the remaining quantity either accumulates in the soil, is lost as run‐off into the surface water or leaches into the groundwater (Steiner *et al*., 2007). Avoiding depletion of soil organic carbon (SOC) and too rapid availability, one can opt for organic fertilizers (Diacono and Montemurro, 2011) typically produced from animal, or plant‐based materials, such as blood meal, feather meal and soybean meal. The decomposition and the rate of nutrient release are based on the activity of soil microorganisms, typically rendering a slower release pattern. Nevertheless, it has been documented that the use of conventional organic fertilizers and soil amendments can lead to the accumulation of heavy metals (HMs), such as copper (Cu), zinc (Zn), lead (Pb) and cadmium (Cd) (Diacono and Montemurro, 2011). Apart from stress on the plants, HM cause detrimental stress on the environment and can potentially be harmful to human health; consequently, novel mitigation strategies should be adopted. Finally, reducing the environmental impact of agriculture would additionally require the reduction of direct (field) and indirect (fertilizer production) emissions, strongly linked to the fertilizer usage efficiency and crop yield.

Microbial biomass presence and/or activity at the soil and rhizosphere can offer advantages at the level of organic matter deposition, stress mitigation and environmental impact. An advantage of using microbes compared to conventional organic fertilizers concerns their production: they can be produced on compact system using recovered resources (Verstraete *et al*., 2016; Pikaar *et al*., 2018). Microorganisms typically have a high nitrogen content, which can slowly be released and fertilize the soil (Pikaar *et al*., 2018). Phototrophic microorganisms have the added value of containing substances that promote plant growth, such as phytohormones and vitamins (Kobayashi and Kobayashi, 1995; Rana *et al*., 2016). Five groups of bacteria are able to carry out photosynthesis: green sulphur bacteria (GSB), green non‐sulphur bacteria (GNSB), purple sulphur bacteria (PSB), purple non‐sulphur bacteria (PNSB) and cyanobacteria. The beneficial effect of using cyanobacteria in plant production has been demonstrated (Coppens *et al*., 2016), while no data were found for the use of GSB or GSNB. In contrast, despite the considerable number of studies on the application of PNSB for plant cultivation, a comprehensive and systematic overview is lacking.

Purple non‐sulphur bacteria, classified as α‐ and β‐proteobacteria, are characterized by high diversity in morphological and physiological characteristics. Their metabolism is unique, as they are able to grow in a variety of cultivation modes (Imhoff, 2006): they can derive their energy from light (phototrophic) under anaerobic conditions as well as from chemical molecules (chemotrophic) under aerobic conditions, both with their carbon source either being derived from CO_2_ (autotrophic) or from organic carbon (heterotrophic). The heterotrophic growth mode results in higher growth rates (1.6–13 day^−1^) (Madigan and Gest, 1979; Rey *et al*., 2006) compared to autotrophic (1.0–8.0 day^−1^) (Madigan and Gest, 1979; Colbeau *et al*., 1980), and infra‐red light can be used as a selectivity tool in the phototrophic growth mode (Hülsen *et al*., 2014), avoiding the growth of algae. This versatility in metabolic functions as well as their tolerance to extreme conditions allows them to grow on a variety of habitats such as (Imhoff, 2006): (i) stagnant water bodies (lakes, coastal lagoons, wastewater ponds, eutrophic ponds) (Kantha *et al*., 2015); (ii) sediments; (iii) moist soils (Kantachote *et al*., 2016; Sakpirom *et al*., 2017); (iv) paddy fields (Kantachote *et al*., 2016; Sakpirom *et al*., 2017); (v) marine environments; (vi) hypersaline environments (Kantachote *et al*., 2016; DasSarma and DasSarma, 2017); (vii) thermal springs and (viii) cold polar habitats. Thus, they are widely distributed in a variety of environments, with the most commonly encountered genera being *Rhodobacter* and *Rhodopseudomona*s (Holguin *et al*., 2001).

Furthermore, PNSB are capable of executing a variety of useful functions such as: (i) nitrogen (N_2_) fixation (Wong *et al*., 2014; Kantha *et al*., 2015); (ii) phosphate solubilization (Koh and Song, 2007; Lee *et al*., 2008; Rana *et al*., 2016); (iii) heavy metal remediation (Batool *et al*., 2017); (iv) methane (CH_4_) emission mitigation (Kantha *et al*., 2015; Sakpirom *et al*., 2017) and (v) CO_2_ sequestration (Tabita, 1995). These traits make PNSB an attractive candidate for multiple applications like use in plant production or as bioremediation agents. In addition, PNSB are known to produce and/or accumulate a diverse range of metabolic products such as: (i) biohydrogen through photofermentative production (Colbeau *et al*., 1980; Koku *et al*., 2002) or during N_2_ fixation (Basak and Das, 2007); (ii) polyhydroxyalkanoates (PHA, including polyhydroxybutyrate PHB) (Melnicki *et al*., 2009; Wu *et al*., 2012); (iii) polyphosphate (Lai *et al*., 2017); (iv) plant growth‐promoting substances (PGPSs) (Nunkaew *et al*., 2014a; Rana *et al*., 2016); (v) carotenoid pigments (e.g. spirilloxanthin, rhodopin, okenone and rhodopinal) (Kobayashi and Kobayashi, 1995; Imhoff, 2006); (vi) siderophores (Sasaki *et al*., 2005); (vii) high amounts of protein (Kobayashi and Kobayashi, 1995); (viii) considerable amounts of essential vitamins (e.g. vitamins B_2_, B_6_, B_12_, C, E, D and folic acid) (Kobayashi and Kobayashi, 1995) and (ix) compounds with health stimulating benefits, for instance reducing LDL‐cholesterol (Ruitang Deng, 2009) or contributing to luminous vibriosis survival (Laranja *et al*., 2014).

This review presents a critical overview of the past and current research regarding the use of PNSB in the context of plant production. First, the key performance indicators (KPIs) of PNSB regarding plant cultivation and their underlying mechanisms are discussed (section Key performance indicators of PNSB for plant production). Next, section Evaluating use of PNSB as fertilizer, bio‐stimulant and bio‐fortifier evaluates and highlights the impact of the PNSB products in plant production, while making a comparison between the use of existing PNSB products (living and dead cells). Section Rice production: harnessing PNSB functionality to its fullest proposes the agricultural application that harnesses the attributes of PNSB at the fullest extent. Finally, this review reveals existing research gaps (section Research gaps and proposed roadmap of PNSB application in plant production) and suggests a roadmap for future research whilst discussing economic aspects.

## Key performance indicators of PNSB for plant production

**Figure 1:**
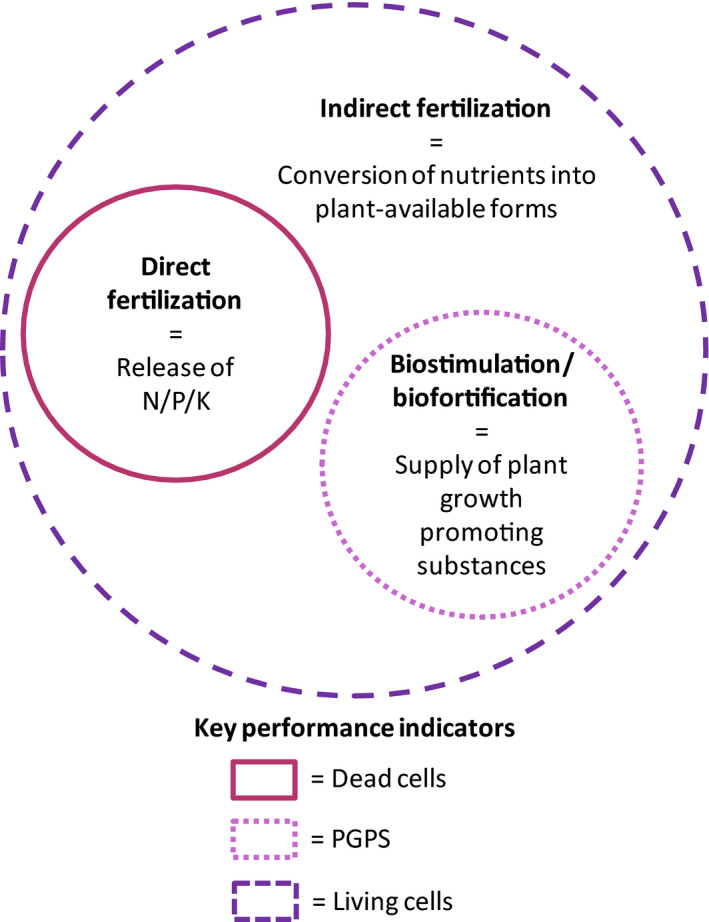
Illustration of the key performance indicators of purple non‐sulphur bacteria (PNSB) in plant production, depending on the type of product used (dead cells, living cells or extracted plant growth‐promoting substance). The functions supported by each PNSB type are the ones contained in the respective boundaries.

Critical analysis of the existing literature revealed three KPIs of each PNSB product, based on different effects on plants (Fig. 1). These include direct and indirect fertilization as well as biostimulation and biofortification. Furthermore, three categories of PNSB products are distinguished, namely living cells, dead cells and cell‐derived products (i.e. PGPS). The KPIs are interwoven with the type of the product as follows: direct fertilization mainly caused by cell decay, therefore, both dead and living cells contribute to this function; indirect fertilization is the result of using living cells where the conversion of nutrients (N and P) into plant‐available forms takes place; and biostimulation and biofortification are the result of the supplied PGPS, where increased resistance to biotic and abiotic stresses is observed. Details about the application method of each PNSB product type can be found in the Appendix S1 (section 1).

**Figure 2:**
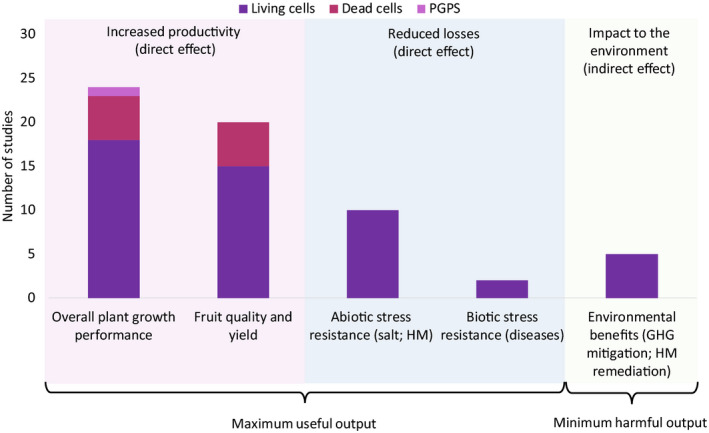
Frequency plot to indicate how many studies in current relevant literature show effects of the purple non‐sulphur bacteria (PNSB) products on the key performance indicators (KPIs) discussed in this review. The frequency plot shows the occurrence of each PNSB product [dead or living cells and extracted plant growth‐promoting substance (PGPS)] in each KPI: overall plant growth performance, fruit quality and yield, abiotic and biotic stresses, environmental benefits. GHG, greenhouse gas; HM, heavy metal.

As shown in Fig. 2, the existing literature mainly focuses on the use of living PNSB cells and less on the use of dead cells, while information regarding the use of extracted PGPS is scarce. Therefore, the latter will not be discussed in the present review (results of all reviewed studies can be found in Appendix S1 in section 2). Furthermore, as can be seen in Fig. 2, there are three distinct effects of the use of PNSB products in agriculture, namely increased productivity, reduced losses due to biotic and abiotic stresses and environmental impacts. The former two concern the useful output of the use of the PNSB products and should be maximized, while the latter aims at the minimization of the harmful environmental output of agriculture. The following section provides an overview of these KPIs of PNSB in agricultural applications.

### Fertilization function

The fertilization function can be divided into two categories: direct and indirect. The former concerns the use of living and dead cells, while the latter is the result of the use of living PNSB cells.

#### Direct fertilization

The use of dead PNSB biomass provides the benefit of a direct process where the only parameter to be considered is the nitrogen/phosphorus/potassium (N/P/K) content, determining the amount of biomass that should be dosed according to the plant's nutrient requirements. This type of microbial fertilizer presents a slow‐release pattern, as the dead microbial biomass is decomposed by the autochthonous soil microorganisms, and the gradually released nutrients are utilized by the plant (Coppens *et al*., 2016). Thereby, synchronous nutrient release and plant nutrient uptake provides superior efficiency (Geng *et al*., 2015). At the same time, such organic fertilizer with PNSB will potentially improve soil structure and stimulate soil microbial activity (Clark *et al*., 1998), resulting in sustainable land usage and a diverse ecosystem (Mäder *et al*., 2002). The same effect is presented when living PNSB cells are used, as the decaying cells can provide N/P/K to the plants. While no literature reports on the full nutrient content of PNSB were found, the N/P/K was measured as 8.5/2.4/0.5% in dry weight (DW) (own data for dried biomass of *Rhodobacter* sp.). Key features include the notably high phosphorus content as compared to microalgae (N/P/K of 8.1/1.3/1.4% DW) (Coppens *et al*., 2016), ascribed to the polyphosphate accumulation (Lai *et al*., 2017), and the relatively low K content that requires supplementation for a balanced fertilization.

#### Indirect fertilization

An additional key functionality of living PNSB cells (compared to dead cells) is the enhanced nutrient availability (Fig. 2), due to microbial activities such as nitrogen fixation and chelation of phosphorus.

##### Biological nitrogen fixation

Many microorganisms can utilize atmospheric nitrogen to support their growth. The microbes associated with nitrogen fixation can roughly be categorized into two groups, symbiotic and free‐living, with PNSB belonging to the latter group (Herridge *et al*., 2008). N_2_‐fixing microorganisms transform atmospheric molecular nitrogen (N_2_), which is biologically unavailable to plants, into ammonia/ammonium (NH_3_/${\text{NH}}_{4}^{+}$), a plant‐available nitrogen form (Franche *et al*., 2009; Olivares *et al*., 2013). The enzyme that catalyses N_2_ fixation is nitrogenase, which is present in all N_2_‐fixing bacteria. There are three types of nitrogenase isozymes, namely molybdenum‐iron (Mo‐Fe), vanadium‐iron (V‐Fe) and iron‐iron (Fe‐Fe) nitrogenases (Sakpirom *et al*., 2017). According to Sakpirom *et al*. (2017), among 235 tested PNSB isolates, *Rhodopseudomonas palustris* TN110 possessed all three different nitrogenase genes and presented the best N_2_ fixation ability. N_2_ fixation by PNSB is most efficient under strict anaerobic conditions, as oxygen inhibits this process (Masepohl and Kranz, 1978). Nevertheless, some PNSB species in the genera *Rhodopseudomonas* and *Rhodobacter* are very tolerant to oxygen and fix nitrogen even under micro‐aerobic conditions (Larimer *et al*., 2004; Hoffmann *et al*., 2014). This largely broadens the application possibilities of PNSB in plant production, for example in paddy fields, which are not strictly anaerobic.

The N_2_ fixation of heterotrophic bacteria can be photo‐dependent or photo‐independent (Pfennig and Trüper, 1989), and the N_2_ fixation ability of PNSB is higher under illumination than under dark conditions (Nunkaew *et al*., 2014a; Wong *et al*., 2014). Therefore, PNSB supply nitrogen more efficiently to the illuminated habitat zones, where nitrogenase activity is not limited by their chemoheterotrophic metabolism as is the case under dark conditions (Harada *et al*., 2005).

Purple non‐sulphur bacteria are distributed in various habitats exposed to sunlight and atmospheric N_2_ (including paddy fields), and their contribution to rendering nitrogen bioavailable to plants has been demonstrated. For instance, when inoculated in a hydroponic nutrient solution lacking a nitrogen source, ammonium was detected in the medium (< 10 μM) (Maudinas *et al*., 1981). Therefore, PNSB being one of the dominant species in paddy fields (Elbadry *et al*., 1999a,1999b) significantly contribute to nitrogen fertility as rice yields can reach values up to 2.0–3.5 ton of nitrogen per hectare using only nitrogen originating from soil organic matter and biological N_2_ fixation (Kundu and Ladha, 1997). Gamal‐Eldin and Elbanna (2011) demonstrated an improved usage efficiency of a synthetic nitrogen fertilizer when a mixture of synthetic fertilizer and *R. capsulatus* was used. At the same time, the use of the mixture resulted in higher grain yield than that obtained from either product (synthetic fertilizer or *R. capsulatus*) when used separately. Biological N_2_ fixation, apart from supplying essential nutrients to plants, also enhances their nitrogen uptake efficiency (Adesemoye *et al*., 2008; Wong *et al*., 2014). For instance, this was validated during rice cultivation in a hydroponic solution inoculated with *R. capsulatus* DSM 155. Specifically, in nitrogen‐deficit medium, the nitrogen content of the root increased by 50–65%, illustrating the N_2_‐fixing abilities of PNSB. On the other hand, when the hydroponic solution contained nitrogen, the effect was less substantial (1.3–14% increase) (Elbadry and Elbanna, 1999). Similarly, the nitrogen content of rice straw rose by 9.2% through seed coating with *R. capsulatus* DSM 155 (Gamal‐Eldin and Elbanna, 2011). The N_2_‐fixing abilities of *R. capsulatus* DSM 155 were also observed when this PNSB strain was inoculated on the roots of rice seedlings, triggering an increase of rice grain nitrogen content by 7.1% (Elbadry *et al*., 1999a,1999b). In this case, the rice straw yield improved by 8.6–24%, with a diminishing effect as the nitrogen content in the fertilizer increased (0–95 kg N ha^−1^). Furthermore, the provision of half the amount of recommended synthetic nitrogen fertilizer in combination with PNSB‐inoculated seeds resulted in rice grain yields statistically comparable to the addition of the full amount of synthetic nitrogen fertilizer (Gamal‐Eldin and Elbanna, 2011). PNSB inoculation could thus result in considerable cost savings due to the reduction of chemical fertilizer use, depending on the required dosage and related production cost for PNSB biomass. Finally, a roughly doubled agronomic nitrogen use efficiency (dry yield per unit of nitrogen supplied) was demonstrated by inoculation with *R. palustris* on pak choi (Wong *et al*., 2014). Similarly, inoculation with *R. palustris* PS3 increased the nitrogen efficiency of lettuce (17%) and pak choi (22–44%) (Hsu *et al*., 2015). Hence, the supply of PNSB can enhance the nitrogen efficiency, potentially contributing to a more sustainable agriculture.

##### Phosphate solubilization

There is evidence that only about 10–20% of the phosphorus applied to agricultural soils is taken up by plants (Schoumans *et al*., 2015). The remaining inorganic phosphorus is adsorbed to clay minerals, as well as iron (Fe^3+^) and aluminum (Al^3+^) ions (at pH < 5.5) or forms crystalline structures with calcium (Ca^2+^) and magnesium (Mg^2+^) ions (at pH > 6) (Schoumans *et al*., 2015). As a result, many agricultural soils are phosphorus‐saturated, creating a phosphorus reservoir (Tóth *et al*., 2014) that shall be exploited. An approach to valorize this reservoir is the application of phosphate‐solubilizing microorganisms (Qian *et al*., 2010). These microorganisms, typically including *Pseudomonas* sp. and *Bacillus* sp. (Sharma *et al*., 2013), can render soil‐bound phosphorus soluble and available to the plants, lowering the need for synthetic fertilizers. Alori *et al*. (2017) suggest that the principal mechanism of phosphate solubilization is the production of mineral dissolving compounds such as organic acids, siderophores, protons (H^+^), hydroxyl ions (OH^−^) and carbon dioxide (CO_2_); which result in pH changes or are active as chelating agents. The ability of PNSB for inorganic phosphate solubilization from soil has been demonstrated by several studies, yet the underlying mechanisms remain unknown (Koh and Song, 2007; Lee *et al*., 2008; Rana *et al*., 2016). Taking into account that the availability of phosphorus can be the limiting step in plant nutrient uptake (Rodríguez and Fraga, 1999), PNSB with phosphate‐solubilizing properties can significantly contribute to an improved plant growth.

*Rhodopseudomonas* sp. produced 64–95 mg l^−1^ soluble phosphate (21–31 mg_P_ l^−1^) from a medium containing 200–800 mg l^−1^ Ca_3_(PO_4_)_2_ (40–160 mg_P_ l^−1^) (Koh and Song, 2007). Koh and Song (2007) reported that the solubilization was low possibly due to the relatively high pH (6.5–7.0) of the medium. Specifically, it has been reported that the optimal pH value for biological phosphate solubilization is around 4.0 (Whitelaw *et al*., 1999); nevertheless, this pH value is not probable to occur in natural plant growing environments (Koh and Song, 2007). Rana *et al*. (2016) tested the solubilization of Ca_3_(PO_4_)_2_, Mg_3_(PO_4_)_2_ and Zn_3_(PO_4_)_2_ by PNSB: *Rhodospirillum rubrum* was able to solubilize these inorganic phosphorus forms with an efficiency of 100%, 100% and 51% respectively. When a mixture of fly‐ash (P‐rich mineral residue of coal combustion) and *R. rubrum* was tested as a fertilizer mixture, the presence of resp. 20, 10 and 4.0 mg l^−1^ of Ca^2+^, Mg^2+^ and Zn^2+^ was observed, indicating the solubilization of phosphate without the release of toxic metal ions (Mn, V, Ni, Cd, As, Hg, B, Cu, Co, Cd, Se, Zn, Mo or Pb) (Rana *et al*., 2016).

### Resistance to stresses

#### Production of plant growth‐promoting substances

Plant growth‐promoting substances are extracellular phytohormones that can be produced by PNSB. They contribute to the coordination of diverse physiological processes in plants including growth and development, as well as in the formation of flowers, leaves, roots, stems, pigments and the development and ripening of fruit (Voß *et al*., 2014). Additionally, they contribute to an increased plant resistance to environmental factors, an induced or suppressed expression of genes and the synthesis of essential compounds such as enzymes, pigments and metabolites (Tsavkelova *et al*., 2006). PGPSs are produced in each cell of the plant and regulate cellular processes in each cell locally as well as in other functional parts of the plant when diffused. Additionally, they are responsible for the differentiation of the cells in each part of the plant (Tsavkelova *et al*., 2006). The pivotal role of PGPS on plant growth has been well established, and lack of these hormones can cause limited and/or abnormal growth (Fahad *et al*., 2015).

The most studied PNSB strains (Tables 1 and 2) in regard to their PGPS production potential are able to produce indole‐3‐acetic acid (IAA) and 5‐aminolevulinic acid (ALA). Melatonin, which is synthesized by some PNSB (e.g. *R. rubrum*) (Manchester *et al*., 1995), may also be a compound of interest as it is considered to be the first‐line defence against oxidative stress in plants (Tan *et al*., 2013). Even though the ability of plants to absorb exogenous melatonin through their roots is proven (Tan *et al*., 2007), its role as a PNSB‐derived PGPS is unexplored to our knowledge and therefore not further discussed in this review.

**Table 1:** Overview of literature on the use of purple non‐sulphur bacteria (PNSB) cell products for the cultivation of several plants (excluding rice). The common name of each plant is presented in bold. All strains were cultivated under photoheterotrophic conditions, unless stated otherwise. Details about the application method and fertilization effect are presented, while details regarding the reference fertilizer and quantitative effect can be found in the Appendix S1

PNSB		PNSB product application modalities			Plant and performance			References
Product type	Strain	Composition and dosage; frequency	Application method	Soil amount	Common name	Species	Effect	
Dead (autoclaved)	*Rhodopseudomonas KL9* and *BL6*	5 × 10^7^ cells; once	Seed inoculation	Soilless cultivation: petri dish	Tomato	*Solanum lycopersicon* Mill. cv. Poongyoung	Increased seedling dry mass	Koh and Song (2007)
Living							Increased germination percentage; increased seedling dry mass; increased seedling length; increased root and shoot length	
Dead (autoclaved)	*Rhodopseudomonas* sp.KL9 and BL6	Suspension^c^ containing 4 × 10^9^ cells; daily for 8 weeks	Soil irrigation	4 kg sand and soil (1:4 v/v)	Tomato	*Solanum lycopersicon* Mill. cv. Zeus	Increased shoot and root dry weight; increased formation ratio of tomato fruit from flower; increased fruit yield; increased fresh weight in harvested fruits	Lee *et al*. (2008)
Living							Increased shoot length; increased shoot and root dry weight; increased fruit‐to‐flower ratio; increased fresh weight and quality (lycopene content) in harvested fruits	
Dead (freeze‐dried)	*Rhodobacter sphaeroides* NR3	2.5 or 1.25 g PNSB; once or split over ten times	Supplied as PNSB powder on soil	15 kg	Tomato	*Solanum lycopersicon* Mill.	Enhanced quality of tomato fruit; increased ascorbic acid content; one‐time application promoted malic acid content; ten‐time application promoted phosphoric acid content	Kondo *et al*. (2010)
Dead (65°C heat‐killed)	*Rhodopseudomonas palustris* PS3, YSC3 and YSC4	50% of standard amount of chemical fertilizer + *R. palustris* strain suspension (1.20 × 10^9^ CFU); weekly for 4 weeks	Soil application	0.3 kg	Pakchoi	*Brassica rapa*s sp. *chinensis*	Insignificant effect	Wong *et al*. (2014)
Living							All PNSB strains: enhanced plant growth; PS3 strain: significant impact on shoot fresh and dry weight; increased fertilizer efficiency; markedly higher plant growth‐promotion rate, especially in poor quality seeds	
Living	*Rhodopseudomonas palustris*	1–5 ml of suspension^b^; every 2 days for 30 days	Foliar spray	1 kg soil containing 1 g fertilizer (N/P_2_O_5_/K_2_O 15/15/15)	Pakchoi	*Brassica rapa ssp. chinensis*	Enhanced photosynthesis; increased crop yield	Xu *et al*. (2016)
Living	*Rhodopseudomonas palustris* PS3 and BCRC16408^d^^,^ e	*R. palustris* suspension^b^ to achieve 3.5 × 10^10^ CFU in 50% Hoaglang solution; weekly for 17 days	Hydroponics	Soilless cultivation	Pakchoi	*Brassica rapa ssp. chinensis*	Improved nitrogen usage efficiency of vegetables; reduced nitrate concentration in the plant	Hsu *et al*. (2015)
		*R. palustris* suspension^b^ to achieve 3.5 × 10^10^ CFU in 50% Hoaglang solution; weekly for 27 days			Lettuce	*Lactuca sativa ssp. Crispa*	Improved nitrogen usage efficiency of vegetables; enhanced plant growth; reduced nitrate concentration in the plant	
Dead (freeze‐dried)	*Rhodobacter sphaeroides* d	0.1 g l^−1^ PNSB cells in 10% Hoagland solution; daily^a^	Soil irrigation	N.A.	Mustard spinach	*Brassica campestris*	Under blue light (470 nm): promoted root growth; increased leaf number; increased chlorophyll and carotenoid contents Under 20% blue (470 nm) – 80% red (660 nm) light: increased average weight of crop; increased leaf number	Kondo *et al*. (2004)
Dead (freeze‐dried)	*Rhodobacter sphaeroides* NR3	0.28; 0.56 and 1.12 g PNSB per pot; once	Supplied as PNSB powder on soil	N.A.	Mustard spinach	*Brassica campestris*	Promoted root growth; increased chlorophyll and carotenoid contents	Kondo *et al*. (2008)
		0.4; 0.8 and 1.6 g PNSB per pot; once			Spinach	*Spinaciaoleracea*	Promoted shoot growth; increased carotenoid content; increased chlorophyll content when sterilized soil was used	
Living	*Rhodopseudomonas* sp. (ISP‐1)	Leaves sprayed with 3.0 × 10^11^ cells; daily for 8 days (total amount of 2.4 × 10^12^ cells); soil irrigated with 3.0 × 10^12^ cells; once (total amount of 3.0 × 10^12^ cells); leaves sprayed with 1.5 × 10^11^ cells; daily for 8 days; and soil irrigated with 1.5 × 10^12^ cells; once (total amount of 2.7 × 10^12^ cells)	Foliar spray: leaves (S); rhizosphere irrigation: soil (I); foliar spray + rhizosphere irrigation leaves and soil (S+I)	N.A.	Stevia	*Stevia rebaudiana*	Enhanced growth; S was more effective; S+I increased: yield; soil dehydrogenase activity, shoot biomass, chlorophyll content in new leaves; and soluble sugar in old leaves were	Wu *et al*. (2013)
Living	*Rhodopseudomonas palustris*	Suspension containing 10^9^ cells; five times^f^	Soil irrigation	Field experiment	Stevia	*Stevia rebaudiana*	Slightly increased leaf dry weight; slightly increased yield	Xu *et al*.(2018)
Dead (autoclaved)	*Rhodopseudomonas palustris* GJ‐22	*R. palustris* suspension to soaking wet (density of 6∙10^7^ CFU ml^−1^); once (seeds); daily for 7 days (leaves)	Seeds; leaves	N.A.	Tobacco	*Nicotianatabacum* L. cv. Samsun NN	Insignificant effect	Su *et al*. (2017)
Living							Increased growth and seed germination; increased root and shoot length; increased plant dry weight; induced virus resistance capability against tobacco mosaic virus	
Living	*Rhodopseudomonas palustris*	Suspension containing 5 × 10^10^ cells; once	Soil irrigation	2.8 kg dry soil	Tobacco	*Nicotianatabacum* L. cv. Yunyan 85	Increased root and shoot dry weight; increased leaf number; reduced As concentrations in rhizosphere soil; reduced As concentrations in root; increased P content in shoot	Hua *et al*. (2014)
Living	N.A.	One litre of 20% (v/v) PNSB cell suspension^b^ water diluted; every 10 days for 3 months	Soil irrigation	N.A.	Mandarin	*Citrus* spp.	Increased number of fruit per tree; increased fruit weight; increased fruit sugar content; increased fruit carotenoid pigments	Kobayashi and Tchan (1973)
Living	N.A.^d^	N.A.	Soil application	N.A.	Persimmon	*Diospyros kaki*	Increased fruit yield; increased fruit quality (sugar and carotenoid content)	Kobayashi and Kobayashi (1995)
Living	Mixed culture of Rhodospirillaceae	Fifty fold diluted culture with OD_652_ = 0.3; twice	Foliar spraying on leaves and young fruit	Field trial	Grape vine	*Vitisvinifera*	Increased fruit‐to‐flower ratio; increased weight per fruit; Increased fruit yield;	Shi *et al*. (1995)
Living	*Rhodopseudomonas palustris*	Four *R. palustris* suspensions: 8.25 × 10^12^; 9.90 × 10^12^; 1.24 × 10^13^ and 1.65 × 10^13^ cells; in four doses	Spraying at casing soil	40 kg compost + 5 cm layer pasteurized soil	Mushroom^g^	*Agaricus bisporus*	Increased mushroom yield; increased number of harvested mushrooms	Han (1999)
Living	*Rhodobacter sphaeroides* Tx25326	*R. sphaeroides* suspension (2.01 × 10^8^ CFU and 1800 ml deionized water); once for 31 days	Soil submerged in suspension	N.A.	Wheat	*Triticumaestivum* L.	Decreased adverse effects from Cd toxicity: decreased Cd exchangeable phases; reduced Cd accumulation in leaves and root (53 and 67% respectively)	Fan *et al*. (2012)
Living	*Rhodopseudomonas palustris*	*R. palustris* suspension containing 2.0 × 10^10^ MPN; twice	Foliar spray (leaves)	N.A.	Chinese dwarf cherry	*Prunushumilis* Bunge	Increased fresh weight and leaf area; increased net photosynthetic rate (Pn); improved antioxidant capacity	Yin *et al*. (2012)
Living	*Rhodopseudomonas* sp. (ISP‐1)	Leaves sprayed with 3.0 × 10^11^ cells; daily for 8 days (total amount of 2.4 × 10^12^ cells); soil irrigated with 3.0 × 10^12^ cells; once (total amount of 3.0 × 10^12^ cells); leaves sprayed with 1.5 × 10^11^ cells; daily for 8 days; and soil irrigated with 1.5 × 10^12^ cells; once (total amount of 2.7 × 10^12^ cells)	Foliar spray: leaves (S); rhizosphere irrigation: soil (I); foliar spray + rhizosphere irrigation leaves and soil (S+I)	N.A.	Stevia	*Stevia rebaudiana*	Enhanced growth; S was more effective; S+I increased: yield; soil dehydrogenase activity, shoot biomass, chlorophyll content in new leaves; and soluble sugar in old leaves were	Wu *et al*. (2013)
Living	*Rhodopseudomonas palustris* CS2; *Rhodopseudomonas faecalis* SS5	Seeds inoculated with PNSB cells suspended in water (0.5 optical density at 600 nm); once	Seeds	N.A.	Bean	*Vigna mungo*	Increased shoot and root length (without and with As stress); increased wet and dry weight (without and with As stress); increased resistance towards As stress	Batool *et al*. (2017)

NA, not available.

Soil irrigation = irrigation of soil with suspension of cells in water.

**a**. Irrigation quantity and frequency not available.

**b**. Amount of cells contained in the suspension not specified.

**c**. Extracted from natural environment.

**d**. Cultivation method not available.

**e**. *Rhodopseudomonas palustris* BCRC16408 did not result in plant performance enhancement.

**f**. Inoculation 60th, 67th, 74th and 81st day after seedling transplanting.

**g**. This mushroom is included in this review since its cultivation is similar to that of plants.

**Table 2:** Overview of literature on the use of purple non‐sulphur bacteria (PNSB) cell products on rice cultivation. All strains were cultivated under photoheterotrophic conditions. Details about the application method and fertilization effect are presented, while details regarding the reference fertilizer and quantitative effect can be found in the Appendix S1

PNSB		PNSB product application modalities			Plant and performance		Reference
Product type	Strain	Composition and dosage; frequency	Application method	Soil amount	*Oryza sativa* L. subspecies and/or cultivar	Effect	
Dead (freeze‐dried)	*Rhodopseudomonas capsulatus*	Amount containing 0.5 g of N, P and K; once during reproductive stage	Supplied as PNSB powder on soil	N.A.	ssp. japonica	Increased rice grain yield	Kobayashi and Haque (1971)
Living	*Rhodopseudomonas capsulatus* B10^1^	*Azotobacter vinelandii* and *R. capsulatus* cells (600 mg and 60 mg protein respectively) in growing medium for rice cultures; once	Seedling roots	Soilless cultivation: hydroponic medium	cv. Delta	Flowering and panicle formation about 100 days after germination (despite the absence of N source in the rhizosphere); enhanced number and size of root hairs	Maudinas *et al*. (1981)
Living	*Rhodopseudomonas capsulatus*	610 kg ha^−1^ compost inoculated with *R. capsulatus* at final concentration of 10^9^ cells g^−1^; twice	Soil application	Field trial	cv. Sasanishiki	Increased rice yield; increased ear number; decreased damage from H_2_S	Yoshida *et al*. (1991)
Living	*Rhodobacter capsulatus* DSM 155^1^	*R. capsulatus* cell suspension in phosphate buffer (pH 7.0) and 10% (w/v) gum Arabic solution; once	Dipping rice seedling for 30 min in cell suspension	N.A.	cv. Giza 176	Increased plant height and dry weight; increased grain yield; increased grain and straw N content	Elbadry *et al*. (1999a,1999b)
Living	*Rhodobacter capsulatus* DSM 155^1^	600 ml nutrients solution inoculated with *R. capsulatus* (final concentration of 2.2 × 10^7^ cells ml^−1^); once	Seedling roots	Soilless cultivation: hydroponic medium	cv. Giza 159; Giza 171; Giza 176 and Giza 181	Increased shoot height; increased shoot dry weight; increased shoot N content; decreased root length and dry weight; increased root number; increased root N content	Elbadry and Elbanna (1999)
Living	*Rhodopseudomonas palustris* KN122	*R. palustris* cells dispersed into sterile distilled water (1st (day 0): 1.7 × 10^11^ cells pot^−1^, 2nd (day 43): 5.0 × 10^9^ cells pot^−1^, 3rd (day 86): 5.1 × 10^10^ cells pot^−1^); once (day 0) or thrice (days 0, 43 and 86)	Inoculated into the floodwater of the pots	0.02 kg or 0.35 kg	cv. Nipponbare	Increased plant height; increased grain yield	Harada *et al*. (2005)
Living	*Rhodobacter capsulatus* DSM 155^1^	*R. capsulatus* cell suspension (10^8^ CFU ml^−1^) in 10% (w/v) gum Arabic solution; once	Seed coating	Field trial	cv. Giza 177	Increased shoot height and weight; increased straw N content; increased number of productive tillers; increased number of grains per panicle; increased grain yield; increased grain N content	Gamal‐Eldin and Elbanna (2011)
Living	*Rhodopseudomonas palustris* TK103, PP803, and P1	One gram of each PNSB product^a^ in 3 l water (without and with salt stress); once	Soil application	0.5 kg	ssp. indica cv. Pathumthani	Reduced inhibition of rice straw and rice husk carrier; increased root dry weight (without and with salt stress); increased root length and shoot dry weight under salt stress; *R. palustris* PP803 increased plant height under salt stress	Kantha *et al*. (2015)
Living	*Rhodopseudomonas palustris* TN114, PP803 and TK103^3^	0.75 kg ha^−1^ of each PNSB product^b^; every 2 weeks during vegetative stage; every week during reproductive and maturation stages	Soil	Field trial	cv. KDML 105 in organic field; cv. RD 41 in saline field) Organic paddy field: only TN114 increased grain yield; decreased CH_4_ flux	Saline paddy field: increased grain yield; increased grains per panicle; decreased CH_4_ flux	Kantachote *et al*. (2016)
Living	*Rhodospirillum rubrum* ATCC 11170	Bacterized according to ISTA protocol; once	Seeds	Field trial	N.A.	Promotion of sprout growth; increased vigour‐index; increased plant survival on fly‐ash; suppressed toxic metal ion release from fly‐ash	Rana *et al*. (2016)
Living	*Rhodopseudomonas palustris* C1, *Rubrivivax benzoatilyticus* C31	PNSB suspension containing 2.5 × 10^11^ cells in rice nutrient broth; weekly refreshed for 30 days	Hydroponics	Soilless cultivation	ssp. indica	Increased root and shoot dry weight; increased shoot height; reduction of As stress; reduction of As accumulation in the plant; enhanced photosynthesis	Nookongbut *et al*. (2018)

NA, not available.

Soil irrigation = irrigation of soil with suspension of cells in water.

**a**. PNSB product preparation: 120 g of mixed carrier (rice straw and husk ash) inoculated aseptically with 30 ml of each PNSB strain (tested individually) and adjusted to final concentration of 10^8^ cells g^−1^ and moisture content of 40% with mature coconut water

**b**. PNSB product preparation: mixed carrier (rice straw and husk ash) containing 18 ml mature coconut water (to achieve moisture content of 40%), inoculated aseptically with 30 ml of each PNSB strain (final concentration of 10^8^ cells g^−1^)

##### Indole‐3‐acetic acid (IAA)

Indole‐3‐acetic acid, or most commonly known as ‘IAA’, belongs to the group of auxins. Auxins are essential for plant development and have a cardinal role in regulating many growth and behavioural processes in the plant's life cycle. They are responsible for plant cell division, extension and specialization (Tsavkelova *et al*., 2006). Specifically, IAA plays an important role in the activation of the cell root and the plant mineral uptake (Sakpirom *et al*., 2017). This PGPS stimulates seed germination and root formation; enhances vegetative growth and fructification; improves photosynthesis, biosynthesis of compounds such as pigments and metabolites; and is responsible for coordinating the plant growth under stress conditions (Tsavkelova *et al*., 2006; Kazan, 2013; Wani *et al*., 2016). Research has shown that IAA aids in the plant adaptation to salinity stress, and enhances the root and shoot growth under salinity and heavy metal stress (Sheng and Xia, 2006; Egamberdieva, 2009; Iqbal and Ashraf, 2010; Fahad *et al*., 2015).

Multiple bacteria have been reported to produce IAA through different biosynthesis pathways, with the indole‐3‐pyruvate and tryptamine pathways identified in *Rhodopseudomonas* sp., while the produced concentrations vary (Spaepen *et al*., 2007). In the study of Sakpirom *et al*. (2017), extracellular production of IAA was found in four PNSB species under micro‐aerobic light conditions yielding 0.65–3.6 mg_IAA_ l^−1^. This variation in produced IAA concentration may lead to a variety of outcomes, ranging from phytostimulation to pathogenesis. For example, the addition of auxin to roots only promotes growth at very low concentrations (0.02–0.18 μg_IAA_ l^−1^), while being inhibitory at higher concentrations (Davies, 1995). This can inhibit root growth, which may result in poor plant development as the root system has a major role in the water and nutrient uptake. Furthermore, PNSB synthesis of IAA was displayed in species isolated from paddy fields and river sediments, as well as insecticide‐tolerant species. The species isolated from paddy fields released IAA in the range 0.65–3.6 mg_IAA_ l^−1^ in their cultivation medium which was supplemented with 1 mM IAA precursor, tryptophan (Sakpirom *et al*., 2017). Similarly, Su *et al*. (2017) found that *R. palustris* GJ‐22 reached an IAA concentration of 30 mg_IAA_ l^−1^ when the tryptophan (3 mM) was added to the culture medium, which was significantly higher than 15 mg_IAA_ l^−1^ when no tryptophan was added. *Rhodopseudomonas* KL9 isolated from river sediments resulted in the highest concentration of 52 mg_IAA_ l^−1^ with 3 mM tryptophan added (Koh and Song, 2007).

##### 5‐aminolevulinic acid (ALA)

5‐aminolevulinic acid, also known as ‘ALA’, is a plant growth regulator. Several studies have focused on the effect of ALA on plant growth (Bindu and Vivekanandan, 1998; Akram and Ashraf, 2013; Nunkaew *et al*., 2014a); however, the mechanisms of ALA‐induced growth and yield promotion have not been elucidated yet. When provided at low concentrations to plants, ALA has plant growth‐promoting properties (Zhen *et al*., 2012; Ali *et al*., 2013). Nevertheless, high ALA levels might cause oxidative stress, thus limiting plant growth (Akram and Ashraf, 2013). Apart from its function as a plant growth promotor, ALA also improves the uptake of minerals and the synthesis of soluble sugars and proteins (Akram and Ashraf, 2013). It is known that ALA is a precursor of the synthesis of chlorophyll, vitamin B_12_, anti‐oxidative enzymes and other metabolites that reduce the adverse effects of various abiotic stress conditions (Wongkantrakorn *et al*., 2009; Nunkaew *et al*., 2014b; Sakpirom *et al*., 2017). Specifically, ALA can increase photosynthesis and therefore promote plant growth at low concentrations of 1–5 mg_ALA_ l^−1^ (Sakpirom *et al*., 2017). Research has demonstrated that ALA can be used in agricultural applications as a compound to boost tolerance towards salinity (Wongkantrakorn *et al*., 2009; Naeem *et al*., 2011; Nunkaew *et al*., 2014b), drought, temperature and low‐light stress in plants (Akram and Ashraf, 2013), as well as a biodegradable herbicide (Sasikala *et al*., 1994; Sasaki *et al*., 2002). Furthermore, ALA has shown to improve the ultrastructure of plant cells, leading to less root damage under stress conditions (Ali *et al*., 2013). The exogenous provision of ALA aids in the accumulation of chlorophyll, resulting in an increase of photosynthetic activity (Bindu and Vivekanandan, 1998; Nunkaew *et al*., 2014a). Finally, ALA enhances the production of ATP and NADPH, which are essential cofactors for CO_2_‐fixation (Sun *et al*., 2009). Since the commercially available ALA is very expensive for most agricultural applications, the use of ALA‐producing microorganisms is viewed as a promising economically viable option for plant cultivation (Nunkaew *et al*., 2014a).

Two ALA biosynthetic pathways are known in bacteria, namely the five‐carbon and the ALA synthase pathway (Avissar *et al*., 1989). In PNSB, the synthesis of ALA is performed by the latter pathway, in a reaction catalysed by ALA synthase that condensates glycine with succinyl‐CoA (Beale, 1990; Sasaki *et al*., 1990). PNSB have been demonstrated to produce ALA under saline or heavy metal stress conditions. For instance, PNSB isolates from paddy fields and Cd/Zn contaminated soils produced 0.23‐5.0 mg_ALA_ l^−1^ under micro‐aerobic light conditions (Kantha *et al*., 2010; Nunkaew *et al*., 2014a; Sakpirom *et al*., 2017). The production of ALA can also be promoted by adding the precursor molecules glycine and succinate to the cultivation medium. In the study of Su *et al*. (2017), concentrations reached 7.9 mg_ALA_ l^−1^ when the precursors were added compared to 4.5 mg_ALA_ l^−1^ in absence of precursors.

#### Mechanisms responsible for abiotic and biotic stress resistance

##### Salinity

Increased salinity has detrimental effects on plant endogenous metabolic processes, such as phytohormone production and photosynthetic activity (Nunkaew *et al*., 2014a). Reactive oxygen species (ROS) are by‐products of aerobic metabolism, formed in the shoot of the plant. These include the superoxide radical anion ($O_{2}^{-}$), hydrogen peroxide (H_2_O_2_) and hydroxyl radical (^·^OH). Their production increases under salinity stress (Ashraf and Harris, 2004), and ROS can have a detrimental oxidative effect if accumulated. Detoxification of ROS occurs through the production of anti‐oxidative enzymes (e.g. ascorbate peroxidase, catalase, glutathione reductase and superoxide dismutase). However, the activity of these enzymes is reduced under extremely saline conditions. Generally, plants producing higher amounts of anti‐oxidative enzymes are more resistant to oxidative damage induced from ROS (De Azevedo Neto *et al*., 2006). In case the plant is unable to produce sufficient amounts of antioxidants, they should either be provided externally, or their synthesis should be promoted. One of these antioxidant promotors is ALA, which acts as a protective mechanism against ROS (Zhen *et al*., 2012). The treatment with ALA protects the photosynthetic apparatus under stress conditions (Sun *et al*., 2009). For instance, rice under salt stress that was pre‐treated with ALA (0.13–0.33 mg_ALA_ l^−1^) presented increased activities of ascorbate peroxidase (126–282%), catalase (950–1067%), glutathione reductase (116–165%) and superoxide dismutase (404–572%) (Nunkaew *et al*., 2014a). Carotenoids are key non‐enzymatic plant antioxidants (Ashraf, 2009). The effect of PNSB on carotenoids varies, ranging from up to 138% increase in spinach provided with dried PNSB cells (Kondo *et al*., 2008), until a 27% decrease in rice cultivated under stress conditions (Nookongbut *et al*., 2018) (for more information refer to Tables S5 and S8). The effect on other important antioxidants, including tocopherols and flavonoids (Ashraf, 2009), is to our knowledge unexplored.

The presence of PNSB in saline paddy fields (3.0–4.0 mS cm^−1^) is an indication that these microorganisms can tolerate saline conditions (Nunkaew *et al*., 2012). Tests with isolated strains indicate that this tolerance can be attributed to the binding of Na^+^ with the extracellular polymeric substances (EPSs) produced by PNSB (Nunkaew *et al*., 2015), and prove the concurrent production of ALA under these conditions (Nunkaew *et al*., 2014a). It has been demonstrated that exogenous supply of low concentrations of ALA (0.01–30 mg_ALA_ l^−1^) can reduce the injurious effect of salinity stress on plants (Wongkantrakorn *et al*., 2009; Naeem *et al*., 2011). Nunkaew *et al*. (2014a) tested two *R. palustris* strains for their ALA production under saline conditions (induced *in vitro* with 0.25% NaCl), and they observed a production of 1180–1705 mg l^−1^ ALA. When cultivated in a saline paddy field, the maximum ALA levels produced by *R. palustris* TK103, PP803 and P1 ranged between 1.4 and 1.7 mg_ALA_ l^−1^, resulting in increased growth and yield parameters (Kantha *et al*., 2015). Inoculation with PNSB presents positive effects on plant growth under saline conditions, specifically on fresh and dry plant and root weight, chlorophyll content and grain yield (Gamal‐Eldin and Elbanna, 2011; Nunkaew *et al*., 2014a; Kantha *et al*., 2015; Kantachote *et al*., 2016), approaching the values recorded for unfertilized plants grown without salinity stress (Nunkaew *et al*., 2014a). The increased root development (Kantha *et al*., 2015) is a great advantage especially under salinity stress, as a high root density can mend the vital functions of rice plants and enhance the grain yield (Mishra and Salokhe, 2011). This ability renders PNSB as eligible for agricultural applications in saline environments.

##### Heavy metals

Purple non‐sulphur bacteria are able to reduce plant stress caused by the presence of HM through a variety of mechanisms, such as accumulation inside the cell, adsorption on EPS bound to the cell's outer surface, conjugation in the siderophores and conversion to less toxic compounds through redox transformations (Panwichian *et al*., 2011; Batool *et al*., 2017; Sakpirom *et al*., 2017; Nookongbut *et al*., 2018). The latter has been demonstrated to be a more effective mechanism for HM stress mitigation in plants than the former two. The speciation of metals in the soil plays a major role, as the exchangeable phases are highly absorbed by the plants (Geebelen *et al*., 2003; Fan *et al*., 2012). At the same time, the accumulation of metals in plants is highly related to their bioavailability. Hence, the reduction of exchangeable species decreases the bioavailability and therefore the inhibiting effect (Fan *et al*., 2012). For instance, the increased root length (33%), when both *R. palustris* CS2 and *R. faecalis* SS5 were supplied (compared to 25–26% for the individual strains), was attributed to the decreased bioavailability of As present in the soil, due to the simultaneous oxidation of As(III) and reduction of As(V) maintaining thus the redox cycle (Batool *et al*., 2017). Finally, Nookongbut *et al*. (2018) argued that the reduction of HM stress to plants by PNSB is a combination of sequestration in siderophores, EPS binding, as well as the enhancement of photosynthesis and activity of antioxidant enzymes through the use of the produced IAA and ALA.

##### Diseases

Plant diseases, viral or microbial, can cause severe damage to crops. The exposure to certain stimuli can enhance the disease resistance potential, and plants that have been exposed to these biotic or abiotic stimuli in the past can rapidly respond to the exposure to a virus by setting off robust defence responses (Conrath *et al*., 2006; Pastor *et al*., 2013). This induced resistance can be catalytic for the plant's survival, especially when triggered prior to infection (Choudhary *et al*., 2007). In this context, PNSB produce IAA, ALA and siderophores, which are compounds that can induce systemic resistance against viruses (Pieterse *et al*., 2014). Foliar fertilization using PNSB has been reported to be efficient in suppressing plant viral diseases such as tobacco mosaic virus (TMV) (Su *et al*., 2017).

### Environmental benefits

#### Heavy metal bioremediation

Although PNSB have been reported to remove metals from wastewaters (Bai *et al*., 2008), they are rarely used for the bioremediation of soil, as they are usually obligate anaerobes and require a lower redox potential (Fan *et al*., 2012). Hence, the best practice is the isolation of strains from environments with conditions similar to the envisaged application. For instance, Fan *et al*. (2012) isolated *R. sphaeroides* from HM‐containing oil field injection water, for bioremediation of Cd from soil. The treatment resulted in a lower Cd concentration in the root and leaves of wheat (67% and 53% reduction resp.) (Fan *et al*., 2012). Moreover, Panwichian *et al*. (2011) tested *Rhodobium marinum* NW16 and *R. sphaeroides* KMS24 for their removal potential regarding HM (Cd^2+^, Cu^2+^, Pb^2+^ and Zn^2+^) from contaminated shrimp pond water under micro‐aerobic, light and aerobic, dark conditions. The results showed that bio‐adsorption by EPS had a greater HM removal efficiency (91–97%) compared to accumulation in the cell (14–75%). Finally, the acid sulphate soil isolates *Rhodopseudomonas* spp. VNW64 and VNS89 reduced Al^3+^ up to 63% and remediated the mixture of Al^3+^ and Fe^2+^ up to 88% (Khuong *et al*., 2017).

#### Greenhouse gas emission mitigation

Apropos of the greenhouse gas (GHG) emissions, plant cultivation can be a net source or a net sink, depending for instance on the balance between methane (CH_4_) emissions from rice cultivation and the CO_2_ uptake by plants (Tubiello *et al*., 2014). In order to reach the longed‐for climate stabilization, GHG sources should be minimized, while carbon sequestration should be maximized (Powlson *et al*., 2011). The potential PNSB contribution to the latter is discussed in section Increased soil fertility.

Purple non‐sulphur bacteria can thrive in environments with micro‐aerobic and anaerobic zones, containing biodegradable compounds. Given that methanogenic archaea grow in similar conditions, these environments are prone to CH_4_ formation. Considering that CH_4_ is a GHG with an effect on global warming 21 times that of CO_2_, the mitigation of CH_4_ emissions is of imperative importance. It is estimated that 26% of the total CH_4_ emissions of 550 Tg year^−1^ originate from wetlands and paddy fields, while the latter contribute to roughly 10% of the total emissions (53 Tg year^−1^) (Cao *et al*., 1998). PNSB have the ability to mitigate CH_4_ emissions in paddy fields by suppressing the growth of methanogenic archaea (Harada *et al*., 2005; Nunkaew *et al*., 2014a; Kantha *et al*., 2015; Sakpirom *et al*., 2017). Given that (i) both methanogens and PNSB compete for the same carbon sources, (ii) PNSB can additionally use CO_2_ as carbon source and (iii) the presence of light gives a great advantage to PNSB, the latter can become dominant in paddy fields (Nunkaew *et al*., 2014b). Several PNSB strains have shown the potential to totally eliminate CH_4_ produced under paddy field conditions, while also reducing the CO_2_ emissions by up to 47% in laboratory scale experiments (Kantha *et al*., 2015; Sakpirom *et al*., 2017). The latter was ascribed to the potential use of CO_2_ as carbon source by PNSB (Kantha *et al*., 2015).

## Evaluating use of PNSB as fertilizer, biostimulant and bio‐fortifier

Critical analysis of the existing literature revealed the different functions of each PNSB product type (Fig. 2), resulting in various effects and interactions with plants. More specifically, the dead cells mainly deliver nutrients, while living cells additionally convert nutrients into plant‐available forms and continuously supply PGPS. This results in the most prominent impact, promoting increased plant growth performance, while suppressing abiotic and biotic stress, as well as reduction of GHG emissions. This section provides a critical comparison of the functionalities of each type of PNSB product.

**Figure 3:**
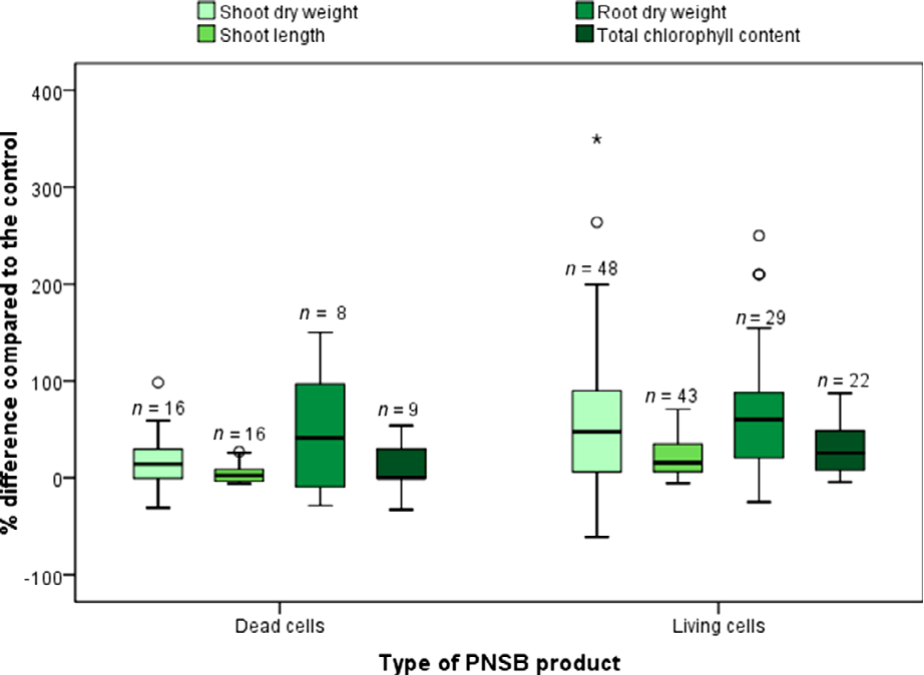
Box plots of the key performance indicators of the studies contained in Tables 1 and 2, regarding the effect of the dead and living purple non‐sulphur bacteria cells on plant growth performance. The effects are given as a negative or positive percentage compared to the control treatment. Above each box plot, the sample size (*n*) is given. The whiskers represent the values within one and a half times the interquartile range, while the individual points are outliers. The values used in the plots as well as information regarding the reference fertilizer can be found in Appendix S1.

**Figure 4:**
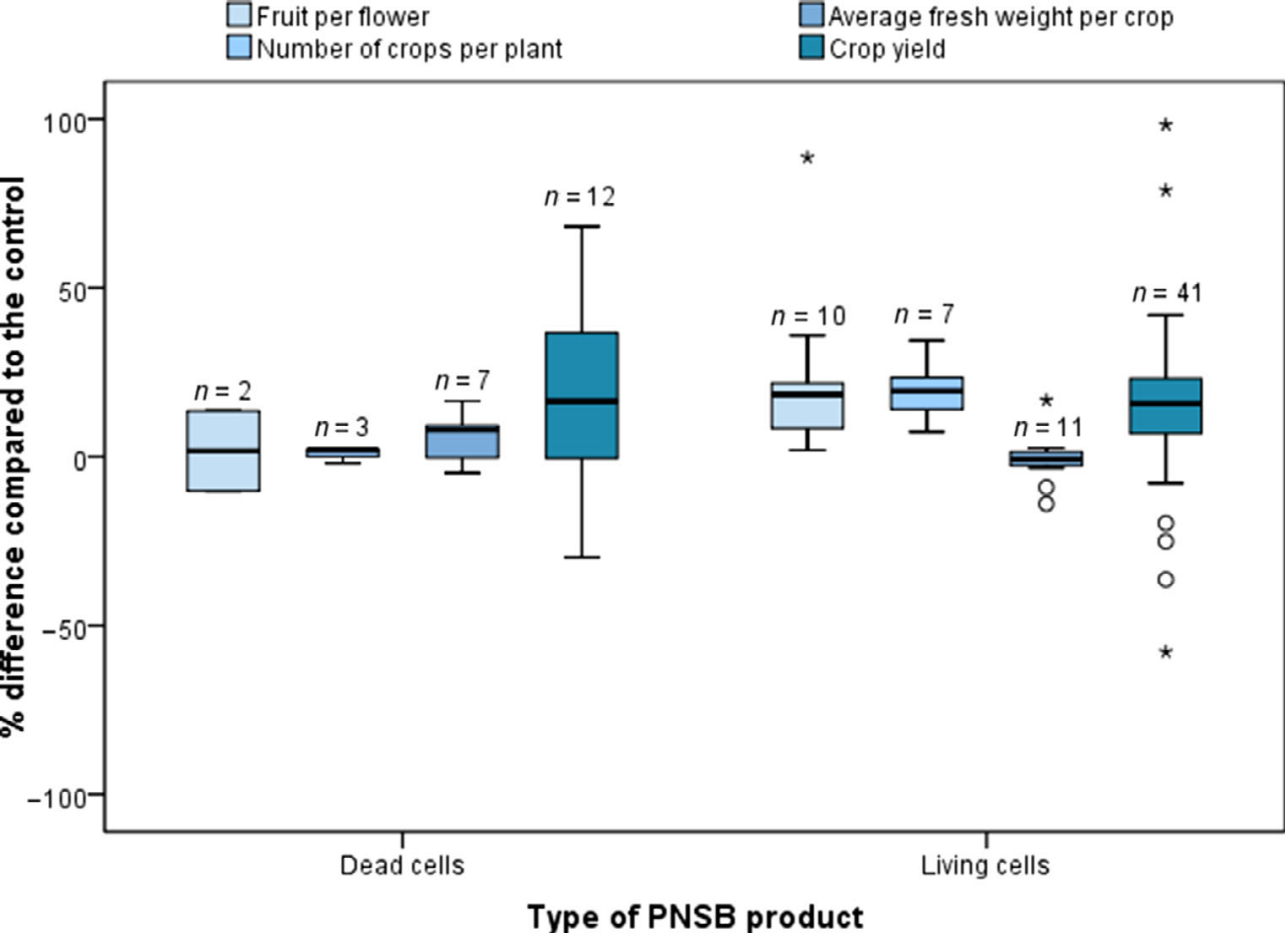
Box plots of the key performance indicators of the studies contained in Tables 1 and 2, regarding the effect of the dead and living purple non‐sulphur bacteria cells on crop yield increase. The effects are given as a negative or positive percentage compared to the control treatment. Above each box plot the sample size (*n*) is given. The whiskers represent the values within one and a half times the interquartile range, while the individual points are outliers. The values used in the plots as well as information regarding the reference fertilizer can be found in Appendix S1.

A systematic analysis and a comparison are made for all results found in literature, and the five major agricultural topics are discussed (Fig. 2). For overall plant growth performance and fruit quality and yield, the comparison is made between the two most occurring PNSB product types: dead cells and living cells. An overview of these data can be found in Tables 1 and 2, and a visual representation is shown in Figs 3 and 4. Over one‐third of the available literature regarding the use of PNSB for plant production concerns rice cultivation (Table 2). The three other topics (abiotic stress resistance, biotic stress resistance and environmental benefits) will only discuss living cells, since no literature was available for dead cells. It should be noted that details about the reference fertilizer, as well as the frequency of fertilization and amount of fertilizer used, can be found in Appendix S1 in section 4. Furthermore, it is recommended to verify the apparent trends as systematically as possible in view of a high methodological variability.

### Fertilization function

This section presents the effects of the use of PNSB products with emphasis on: (i) overall plant growth performance showing the effects on the whole plant, including shoot and root and (ii) yield and quality of the edible plant biomass, where the effect on crop/produce is discussed. It should be noted that some of the observed effects might not be strictly due to fertilization (nutrient supply) but biostimulation and/or biofortification can have contributed to the result. This distinction is made to facilitate the understanding of the effects on the different categories.

#### Overall plant growth performance

The comparison of dead and living cells for the different KPI within plant performance is illustrated in Fig. 3. For overall plant growth performance, four KPIs were chosen: shoot dry weight, shoot length, root dry weight and total chlorophyll content. For all KPI, mostly positive effects are seen compared to the control. Overall, the living cells show a larger variability in the positive direction compared to the dead cells, while the median is also higher for each KPI for living cells, meaning that more than 50% of the results were more positive compared to dead cells.

In accordance with the meta‐analysis showing that living biomass had a more positive effect on plant growth performance, tests under the same crop growth conditions showed a better performance of dosing an equal amount of living biomass. Specifically, when Koh and Song (2007) inoculated with living cells of *Rhodopseudomonas* sp. BL6 and KL9 to tomato seeds, they observed a 7.6–32% increase in germination percentage, as opposed to the application of autoclaved cells. This showed that germination is more affected by bacterial metabolism than by plant‐available nutrients contained in the bacterial cells (Koh and Song, 2007). The positive effects of PNSB metabolites on plant growth were further observed during hydroponic tests. Shoots of rice seedlings cultivated in a nutrient solution inoculated with *R. capsulatus* DSM 155 were up to 75% taller, and significantly heavier (up to doubled dry weight) compared to uninoculated controls (Elbadry and Elbanna, 1999). Moreover, the addition of living PNSB had notably better effects on tomato plant growth compared to the same amount of autoclaved cells (47–121%, 18–35%, 12–79% increase in shoot dry weight, shoot length and root dry weight as compared to 2.8–34%, 0% and 0–26% for autoclaved biomass) (Lee *et al*., 2008). Heat‐killed or autoclaved cells of *R. palustris* did not display any notable effects on the growth of pak choi at soil application (Wong *et al*., 2014), nor on the growth of tobacco during soil application (Hua *et al*., 2014) or foliar fertilization (Su *et al*., 2017). In contrast, the same amount of living cells presented significant positive effects on plant growth (Hua *et al*., 2014; Wong *et al*., 2014; Su *et al*., 2017) (see sections 3.1 and 3.2 in Appendix S1).

The effectiveness of dead cells is attributed to the fact that their use promotes the activity of soil microbes which use the PNSB biomass nutrients for their own growth, therefore, demonstrating similar properties with organic fertilizers. This was indicated by Kondo *et al*. (2008), where the use of sterilized soil resulted in a lower increase in fresh and dry shoot weight, as well as length and carotenoid content of spinach, compared to the use of non‐sterilized soil. Therefore, it can be concluded that the use of dried PNSB promotes the activity of soil microbes, which produce elevated amounts of PGPS (Kondo *et al*., 2008), indirectly displaying the effects of living PNSB biomass.

#### Yield and quality increase of edible plant biomass

The use of PNSB enhances both quantity and quality of harvested crops. The effect on the yield and quality of edible plant biomass was also quantified based on four KPIs: fruits per flower, number of fruit/grain/edible leaves (the more general term ‘crops’ is used for conciseness) per plant, average fresh weight per crop and crop yield (Fig. 4). The majority of the results have positive outcome when dead or living PNSB cells were dosed compared to the control. For instance, autoclaved *Rhodopseudomonas* sp. BL6 and KL9 cells significantly promoted the average tomato fruit yield (42–50% increase) while inoculation with the same amount of living BL6 and KL9 enhanced the yield by 21–98% (Lee *et al*., 2008). Furthermore, there is smaller variability between the outcomes compared to the results for overall plant growth (Fig. 3), with the exception for the crop yield, which also shows more extreme outliers for living cells. There is no straightforward trend showing an overall better performance of dead or living PNSB cells regarding KPI for edible plant biomass.

A general remark is that, in most cases, the yield is increased by the elevated number of fruits or grains rather than a higher individual crop weight. For instance, Kobayashi and Kobayashi (1995) reported a 16% rise in persimmon fruit yield due to 34% more fruits, whereas the average weight per fruit decreased by 14%. Elbadry *et al*. (1999a,1999b) demonstrated a 30% higher rice grain yield, but the weight of individual grains decreased by 9.1%. This indicates that PNSB products enhance the new grain formation rather than the individual grain weight increase, as shown by the 3.9% rise in the number of grains per panicle (Elbadry *et al*., 1999a,1999b). An increase in grain yield was observed in all cases (19–33%), attributable to the higher number of productive tillers, with a most notable effect when nitrogen fertilizer was not provided (Elbadry *et al*., 1999a,1999b). Han (1999) ascribed the increased numbers and yield of mushroom1Mushrooms are included in this review since their cultivation is similar to that of plants. (7.4–26% and 10–22% resp.) to the enhanced nutrient provision as well as to the ALA contained in the suspension. No significant effect was observed regarding the size of the mushrooms, with the latter indicating that the PNSB stimulated the formation of more primordia (cells in the earliest stage of development). In another study, inoculation with *R. palustris* KN122 improved the rice grain yield (8.9–24%), whereas the grain weight was not affected by the treatment (Harada *et al*., 2005). Nevertheless, there is a limited number of studies reporting elevated fruit weight (Kobayashi and Tchan, 1973; Shi *et al*., 1995; Lee *et al*., 2008). Specifically, the use of PNSB biomass resulted in higher number of mandarin fruits per tree (9.1%), fruit yield (27%), as well as the average weight per fruit (17%) compared to the control (Kobayashi and Tchan, 1973). When Shi *et al*. (1995) used foliar spray containing mixed PNSB cultures on grapes, they observed a 2.5% rise in the average fruit weight. More importantly, the ratio of fruits per flower increased by 1.9%, which is important as the flowers of this plant commonly die due to weakness of the plant and nutrient limitation (Shi *et al*., 1995). Similarly, Lee *et al*. (2008) observed that the provision of *Rhodopseudomonas* sp. KL9 doubled the weight of harvested tomatoes, while the formation ratio of tomato fruit from flower also increased (14–89%).

It has been demonstrated that PNSB, apart from increasing the quantity, can also improve the quality of the harvested edible plant biomass. It was demonstrated that dead PNSB cells perform equally as well as conventional inorganic fertilizers ((NH_4_)_2_SO_4_) in terms of tomato quality increase (Brix sugar content, titrable acidity, carotenoid and citric acid content) (Kondo *et al*., 2010). Moreover, Kobayashi and Tchan (1973) reported that the use of living PNSB resulted in sweeter mandarin fruit (5.8% higher sugar content) and more attractive visual appearance due to 20% more carotenoids. The taste as well as the appearance of persimmon fruit improved, as indicated by an elevated sugar and carotenoid content (15% and 20% increase resp.) (Kobayashi and Kobayashi, 1995). Similarly, the use of *R. sphaeroides* NR3 enhanced the carotenoid content of spinach (14–138%) and mustard spinach (4.1–21%) (Kondo *et al*., 2008). Lee *et al*. (2008) concluded that a significant increase in lycopene content (1.7–48%) of tomato fruit was the result of the stimulation of tomato plant metabolism from the symbiosis with *Rhodopseudomonas* sp. BL6 and KL9. Additionally, the use of *Rhodopseudomonas* sp. (ISP‐1) resulted in 77–116% more soluble sugars in stevia leaves, 30–91% rise in chlorophyll a and 29–82% chlorophyll b, while the stevioside content rose up to 69% (Wu *et al*., 2013). The nitrogen content of rice grains was 7.1% higher due to the inoculation with *R. capsulatus* DSM 155 (Elbadry *et al*., 1999a,1999b). Finally, inoculation with *R. palustris* PS3 reduced the nitrate content of the nitrate‐rich vegetables pak choi (20–50%) and lettuce (27%) (Hsu *et al*., 2015). This could have a potentially positive effect on high nitrate diets (e.g. Mediterranean, Japanese) since the high dietary nitrate intake is often associated with health risks (Lidder and Webb, 2013).

### Resistance to stress

This section discusses the enhanced resistance to stresses (abiotic and biotic), induced by the supply of living PNSB cells.

#### Resistance to abiotic stress

Research shows that the use of PNSB in plant cultivation can reduce the yield losses due to abiotic stresses and/or diseases. For instance, the lack of light during the dark months negatively affects plant growth and crop yield. Kondo *et al*. (2004) noted that the use of dried *R. sphaeroides* can compensate for the lack of the full light spectrum on the growth of mustard spinach. It was observed that the average weight of the vegetable increased by 17% during the 20% blue – 80% red light treatment, while the crop quality significantly increased with the PNSB product under blue light as indicated by the elevated chlorophyll a (61%) and chlorophyll b (39%) content. Finally, Wong *et al*. (2014) found that the effect of living PNSB fertilization on old seeds of pak choi was higher than on new seeds, indicating that this is a good technique to avoid the costly damage of stored seeds.

The adverse effects of salinity stress on plants have been successfully minimized by applying living PNSB, as demonstrated with experiments using rice plants. For instance, coating with *R. capsulatus* DSM 155 resulted in 18–33% higher rice grain yields (*Oriza sativa* L. cv. Giza 177) during cultivation in a saline paddy field (Gamal‐Eldin and Elbanna, 2011). At the same time, the use of living PNSB in saline paddy fields has been reported to enhance all plant growth parameters (Kantha *et al*., 2015). Specifically, Kantha *et al*. (2015) inoculated *R. palustris* TK103, PP803, and P1 in rice plants (*Oryza sativa* L. subsp. indica) under salt stress. The root dry weight and length as well as the shoot length and dry weight increased (210–250%, 80–105%, 36–45% and 73–115% resp.). *R. palustris* PP803 most efficiently limited the negative effects of salinity stress on rice plants (highest increase for all growth parameters). Similar results were reported by Kantachote *et al*. (2016), where during the cultivation of rice in saline fields, the shoot height and panicle weight increased by 4.1–10% and 21–33%, while the number of rice grains per panicle and rice grain yield were elevated (8.4–18% and 5.2–9.0% resp.) using a mixed carrier inoculated with *R. palustris* TN114, PP803 and TK103 in a saline paddy field (*Oriza sativa* L. cv. RD 41). These results indicate that under salinity stress, PNSB fertilization enhances the grain production rather than the plant growth (Kantachote *et al*., 2016).

Stress due to the presence of metals can also be mitigated using living PNSB, as reported in experiments performed on wheat and bean plants. Fan *et al*. (2012) treated Cd‐contaminated soil with *R. palustris,* and the Cd bioremediation efficiency was tested by planting wheat seeds (*Triticum aestivum* L.). No significant improvement of plant growth was observed, with the level of inhibition being a function of the Cd concentration; however, the Cd accumulation in roots and leaves decreased by 67% and 53% resp. (Fan *et al*., 2012). Batool *et al*. (2017) used the As‐resistant *R. palustris* CS2 and *R. faecalis* SS5 on bean plants (*Vigna mungo*) cultivated in As‐contaminated soil. *R. palustris* CS2 and *R. faecalis* SS5 were able to tolerate resp. 150 and 100 mM As(V) and Cr, Ni and Zn at a concentration of 1.0 mM, while they could not tolerate Cu, Cd and Co. *R. palustris* CS2 reduced As(V) to As(III) whereas *R. faecalis* SS5 oxidized As(III) to As(V). It should be noted that As is mainly found in two forms: arsenate (As(V)) and arsenite (As(III)) (Oremland and Stolz, 2003), with the latter being roughly 100 times more toxic compared to As(V) (Jain and Ali, 2000). *R. palustris* CS2 presented the highest As(V) reduction potential of 63%, while SS5 showed the highest As(III) oxidation potential of 96% (providing 10 mM As(V) and 5 mM As(III)). A significant enhancement of growth was observed in plants inoculated with the individual PNSB, under As exposure (25–26% and 31–33% increase in root and shoot length), while the effect was greater when a mixture of both strains was used (33% and 37% increase resp.). Moreover, the inoculation with *R. palustris* of tobacco plants cultivated in As‐contaminated soil increased shoot height (14%), as well as root and shoot dry weight (32% and 6.8%) (Hua *et al*., 2014). Furthermore, the yield increased as shown by the elevated leaf number (20%) and leaf dry weight (42%). Importantly, the As concentration of the plant decreased by roughly 15% with the largest effect presented in the root concentration (43% decrease), however, the As concentration in the leaves was not significantly affected. Similarly, *R. palustris* C1 and *Rubrivivax benzoatilyticus* C31 reduced the accumulation of As in rice plants (*Oriza sativa* ssp. *indica*) while the mixture of strains enabled the highest reduction (up to 65%) (Nookongbut *et al*., 2018). Most importantly, even though the control plants contaminated with a mixture of As(III) and As(IV) as well as the ones inoculated with the individual strains did not survive, the mixture of PNSB enabled their growth. This potentially renders the synthetic community suitable for real application in fields where both As species exist.

#### Resistance to biotic stress

Living PNSB have the capacity to suppress plant diseases, as demonstrated through experiments with rice and tobacco plants (Tables 1 and 2). Specifically, this was validated by Rana *et al*. (2016) who reported that *R. rubrum* resulted in total elimination of disease incidence in rice (*Oriza sativa* L.). Similarly, during experiments with tobacco plants, Su *et al*. (2017) used *R. palustris* GJ‐22 foliar spray against of tobacco mosaic virus (TMV), which is registered as one of the ‘top 10’ economically important plant viruses (Rybicki, 2015). This treatment presented similar performance to a commercially available disease resistance inducer [i.e. BTH or benzo (1,2,3) thia‐diazole‐7‐carbothioic acid S‐methyl ester]. Specifically, under axenic conditions, *R. palustris* GJ‐22 colonized the phyllosphere of tobacco plants (*Nicotiana tabacum* L. cv. Samsun NN) and exhibited enhanced virus‐resistance‐inducing capacity against TMV, while the produced PGPS resulted in enhanced seed germination (21%), growth performance and resistance to TMV. Likewise, under field conditions, the PNSB foliar spray enhanced the activities of defensive enzymes and protected the tobacco plants against TMV. Specifically, the use of *R. palustris* GJ‐22 presented similar results to the provision of BTH, with 74% and 70% lower TMV accumulation for BTH‐ and GJ‐22‐treated leaves. GJ‐22 cells increased the yields by 30–32% while BTH resulted in lower values (10–12%). The first‐class tobacco leaves increased by 28–40% with GJ‐22, whereas the BTH spray enhanced the yield of leaves by roughly 23%. The disease severity lowered to resp. 12–13% and 11–12% with GJ‐22 and BTH, while the corresponding values were 25–26% for the control (water). It should be noted that the same amount of autoclaved *R. palustris* GJ‐22 did not present any significant effect regarding growth parameters and resistance to TMV (Su *et al*., 2017). Finally, it was demonstrated that the inoculation of *R. faecalis* increased the antifungal activity of several strains belonging to the *Bacillus* genus against the root rot fungus *Helicobasidium mompa* using disk placement tests (We *et al*., 2016).

### Environmental benefits

#### Sustainability of PNSB production

A superiority of PNSB products compared to conventional fertilizers is the possibility of utilizing inexpensive feedstock and/or waste(water) as sources of carbon and nutrients to produce biomass (Nasseri *et al*., 2011). This means that PNSB products can be produced on nutrient‐rich agro‐industrial or process liquid side‐streams (e.g. potato cutting‐waters). These side‐streams are often treated without generating a recovery product with distinctive value and/or demand, further contributing to an increased pressure on natural resources. The pressure on P, for example, has been steadily enlarging (Verstraete *et al*., 2016). Phosphate fertilizers are made from apatite, a group of phosphate minerals identified as one of the 20 critical raw materials in Europe (European Commission, 2017). More specifically, apatite is expected to be depleted in 50–100 years if the rate of consumption remains the same as nowadays (Cordell *et al*., 2009). Finally, given that the microbial product is entering the food chain only indirectly (i.e. use as fertilizer), system operation in strictly axenic conditions is not required therefore facilitating the overall handling (Pikaar *et al*., 2018).

#### Increased soil fertility

The maintenance of soil fertility is important for sustainable land use, supply of nutrients that are vital for plant growth and the stimulation of the active and diverse ecosystems (Clark *et al*., 1998; Mäder *et al*., 2002). This results in an appropriate soil structure and permits the organic material decomposition through natural processes (Mäder *et al*., 2002). PNSB products supply the soil with nutrients in their organic form, resulting in the enhancement of soil quality, through the provision of SOC, in contrast to synthetic fertilizers. Given that the intensification of agriculture has dramatically decreased the SOC content of agricultural land (Lal, 2004), PNSB products can contribute to the restoration of soil quality. Finally, PNSB are able to convert atmospheric CO_2_ into biomass (Kantha *et al*., 2015) and can contribute to the targeted yearly 0.4% increase in soil carbon stocks, which is expected to contribute to food security and climate stabilization (Vermeulen *et al*., 2019).

#### Heavy metal bioremediation

There is evidence that PNSB have the ability to resist and bioremediate HM, including Cd, Zn, Al, Fe and As. This ability was demonstrated by Sakpirom *et al*. (2017), where *R. palustris* TN110 and *Rubrivivax gelatinosus* TN414 were isolated from Cd and Zn contaminated paddy fields. *R. palustris* TN110 removed resp. 84% and 55% of Cd and Zn (initial concentration of 262 mg l^−1^), while for *R. gelatinosus* TN414, the removals reached the values of resp. 72% and 74% (initial concentration of 23 mg l^−1^). The HM resistance of these strains (estimated by comparing growth on a biotic control) was resp. 68% and 41% for *R. palustris* TN110 and 65% and 51% *R. gelatinosus* TN414 for Cd and Zn (Sakpirom *et al*., 2017). Fan *et al*. (2012) tested the Cd bioremediation efficiency of *R. palustris* on Cd‐contaminated soil. The treatment did not reduce the Cd concentration, but the oxidation‐reduction potential decreased. Hence, Cd was converted to more stable, less plant‐available forms. On the other hand, *Rhodopseudomonas* spp. VNW64 and VNS89 remediated Al^3+^ 42–63% and a mixture Al^3+^ and Fe^2+^ 59–88% through biosorption, showing great potential as acid sulphate soil bioremediation agent (Khuong *et al*., 2017). Similarly, the inoculation with *R. palustris* decreased the concentration of total As in rhizosphere soil by 24% (Hua *et al*., 2014).

#### Greenhouse gas emission mitigation

It has been demonstrated that PNSB can contribute to a more sustainable agriculture, through reducing the GHG emissions. More specifically, PNSB have shown the potential of mitigating CO_2_ emissions by 33–47%, through the use as carbon source for their growth, while reducing the CH_4_ emissions by 100% (Kantha *et al*., 2015; Sakpirom *et al*., 2017). This high potential for CH_4_ mitigation in paddy fields is attributed to the fact that PNSB can proliferate and outcompete native methanogenic microbes in saline fields with HM contamination (Sakpirom *et al*., 2017). Specifically, Harada *et al*. (2003) incubated paddy field slurries combined with rice straw under anaerobic conditions in individual tests inoculated with 16 isolated *R. palustris* strains and observed a 44–62% CH_4_ reduction. In similar experiments, *R. palustris* PP803 and TN114 suppressed CH_4_ by resp. 88% and 70% (Nunkaew *et al*., 2014a). Kantha *et al*. (2015) inoculated *R. palustris* strains PP803, P1 and TK103 in soil from paddy field and ground rice straw under salt stress. CH_4_ emissions reduced resp. by 100%, 94% and 86%, while CO_2_ decreased by 47%, 44% and 38%, using strains PP803, P1 and TK103. Finally, Sakpirom *et al*. (2017) investigated the potential of PNSB to suppress the growth of native paddy field methanogenic bacteria under simulated paddy field conditions. The inoculated PNSB strains showed better performance than the autochthonous, with *R. palustris* TN110 presenting the highest reduction of 80% and 33% for CH_4_ and CO_2_ respectively.

## Rice production: harnessing PNSB functionality to its fullest

**Figure 5:**
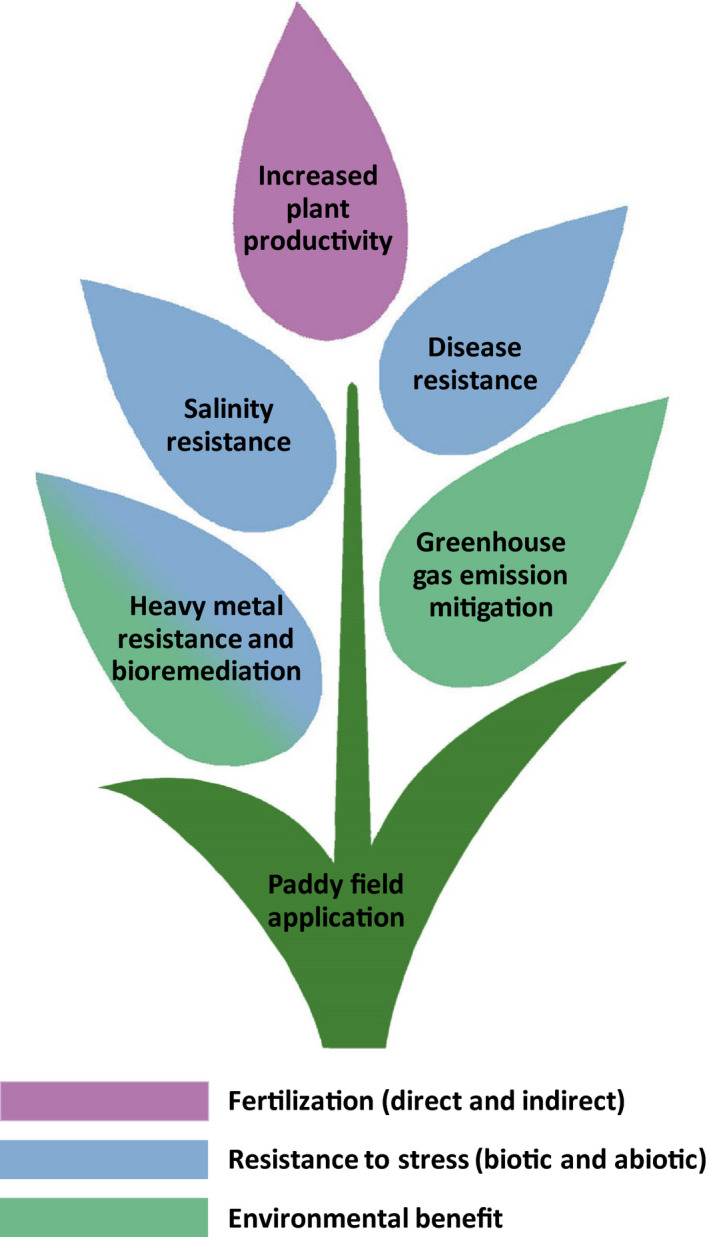
Summary of the beneficial functionalities of applying living purple non‐sulphur bacteria for plant production, hinting at paddy field application as most promising to harness all effects.

As discussed in the previous section, PNSB have the ability to enhance plant growth performance, increase crop yield and quality, reduce the adverse effects of salinity and HM stress, increase the resistance of plants towards diseases, bioremediate HM and mitigate GHG emissions (Fig. 5). The combination of these effects leads to the most promising application, being the use in paddy fields for rice cultivation. Growth performance enhancement, crop yield and quality increase, as well as disease resistance are effects non‐exclusive to paddy fields, and have been thoroughly elaborated in sections Fertilization function and Resistance to biotic stress. Therefore, they will not be discussed here. It should be stressed though, that there is more than one capability of PNSB enabling these effects. For instance, Kantachote *et al*. (2016) concluded that the increased rice plant growth performance (cultivated in paddy field) could be attributed to (i) the lower concentration of H_2_S, which is an inhibiting compound for plant growth due to its use as electron donor by some PNSB strains (Harada *et al*., 2001b), in addition to (ii) N_2_ fixation, as well as (iii) the production of phytohormones.

It is of key importance that paddy fields provide favourable conditions for PNSB growth, due to the combination of an anaerobic environment containing acetate which originates from rice straw degradation (reaching mM levels) (Conrad, 2007) and sunlight. In the study of Kantachote *et al*. (2016), all PNSB strains proliferated in both organic and saline fields, as indicated by the increase of 1 log CFU g^−1^ compared to the control. This gives PNSB the advantage of potentially outcompeting native microbes and allotting their positive effects on rice plants throughout their growth. It should be noted that PNSB are inherently present in paddy fields, nevertheless, their number depends on the field conditions (Elbadry *et al*., 1999a,1999b). Specifically, Elbadry *et al*. (1999a,1999b) reported that PNSB populations increase after rice transplanting, reaching their maximum numbers at tillering stage and declining until the time of harvest. It is therefore suggested here, that PNSB inoculation at an early stage of the rice production cycle can result in beneficial for rice production effects. An interesting remark is that PNSB inoculation had a more prominent effect on the (PNSB) population contained at the outer soil layer (0–1 cm depth) in the case where rice straw was not applied (rice straw application is a common practice to enhance soil fertility during rice cultivation) (Harada *et al*., 2005). Specifically, in the study of Harada *et al*. (2005), the uninoculated control had PNSB populations of 10^4^–10^5^ most probable number (MPN) g^−1^ soil DW whereas the inoculation resulted in 10^8^ MPN g^−1^ soil DW. On the other hand, the uninoculated control containing rice straw had PNSB populations comparable to that of inoculated pots (10^7^ and 10^8^ MPN g^−1^ soil DW respectively). These results illustrate that, when straw is applied, PNSB populations naturally increase around the surface soil. As indicated by the larger PNSB populations on bulk soil (4–6 cm) in the latter case (10^5.5^–10^6^ compared to 10^5^–10^5.1^ MPN g^−1^ soil DW), when PNSB are inoculated under these conditions, part of the community moves in the deeper soil levels resulting in a more prominent positive effect on plant growth enhancement (Harada *et al*., 2005).

Furthermore, when similar experiments were performed under non‐saline and saline conditions, the increase in growth and yield parameters was higher under salinity stress, indicating a beneficial effect of produced phytohormones under these conditions (Gamal‐Eldin and Elbanna, 2011; Kantha *et al*., 2015; Kantachote *et al*., 2016). This is a promising ability, especially considering that saline fields are annually increasing by 10%, with 20% of the worldwide cultivated land already suffering from high salinity (Shrivastava and Kumar, 2015). Kantachote *et al*. (2016) noted that there was no difference in PNSB populations between organic and saline fields, indicating that the binding of Na^+^ from the produced EPS may be an effective mechanism to increase PNSB survival in saline soil.

Several experiments revealed the ability of PNSB to bioremediate HM such as Cd, Zn (Panwichian *et al*., 2011; Sakpirom *et al*., 2017), Al, Fe (Khuong *et al*., 2017) and As (Batool *et al*., 2017). Cd and Zn are commonly found in areas with intensive mining (Li *et al*., 2014; Xu *et al*., 2014). Taking into account that paddy fields close to the aforementioned areas are still in use (Xu *et al*., 2014), the concentrations of these HM are building up. Al and Fe are basic components of acid sulphate soils which are used for rice cultivation in Asiatic countries (Panhwar *et al*., 2016). This type of soil causes drastic reduction of rice yields, mainly due to metal toxicity, and are expected to have a major effect in the future food security. Furthermore, As is a toxic HM naturally occurring or released by industrial processes. When plants are grown in As‐contaminated waters, the growth and yields are reduced as a result of toxicity (Li *et al*., 2007). As‐resistant PNSB have the ability to oxidize and reduce As in addition to adsorb and desorb it, as a defence mechanism against As toxicity (Stolz *et al*., 2006; Nookongbut *et al*., 2016). It is recognized that paddy fields are amongst the areas that suffer the most from increasing As accumulation (Meharg and Rahman, 2003). Therefore, rice cultivation in paddy fields is often limited due to HM contamination, and thus, PNSB can play an important role in their bioremediation.

Paddy fields have been identified as one of the major contributors of CH_4_ emissions (Yagi and Minami, 1990). Specifically, it is estimated that about 10% of the total annual CH_4_ budget originates from rice cultivation (Cao *et al*., 1998). This CH_4_ source is particularly important, as the rice produced in paddy fields is a major component of the global economy and nutrition. CH_4_ production in paddy fields is enhanced by the addition of straw, which is a common technique to increase soil fertility in flooded paddy fields (Harada *et al*., 2005; Nunkaew *et al*., 2014a; Kantha *et al*., 2015; Sakpirom *et al*., 2017). Straw is metabolized to acetic acid by anaerobic microbes and is subsequently converted to CH_4_ (Conrad, 2007). Studies performed under mimicked paddy field conditions prove the potential of PNSB to suppress the growth of methanogens in paddy fields (Harada *et al*., 2003; Nunkaew *et al*., 2014a; Kantachote *et al*., 2016; Sakpirom *et al*., 2017). As explained in section Greenhouse gas emission mitigation, due to their suitability for PNSB growth, paddy field conditions may result in higher growth rates for PNSB compared to methanogens, enabling them to eventually outcompete the methanogens (Harada *et al*., 2001a). Moreover, PNSB have the ability to reduce CO_2_ emissions (Kantha *et al*., 2015; Sakpirom *et al*., 2017), even when paddy fields are characterized by high salinity or are contaminated with HM. The rechanneling of organics into PNSB biomass (rather than CH_4_ and CO_2_) was demonstrated during tests with paddy soil incubated with rice straw under illuminated conditions, where the MPN of PNSB cells reached values of 1.0∙10^10^ MPN g^−1^ soil DW whereas the corresponding value for unilluminated assays was 4.0∙10^8^ MPN g^−1^ soil DW (Harada *et al*., 2005).

## Research gaps and proposed roadmap of PNSB application in plant production

Even though the use of PNSB appears to be a promising approach for many applications, there is still a plethora of questions to be answered. This section discusses the key research gaps that emerged from reviewing existing literature and proposes a roadmap for future research and implementation. It should be stressed that the analysis of the existing literature revealed the need for a more comparable design of experiments and standardized measurement of key parameters, to facilitate systematic comparison and more generically valid findings.

### Research, development and demonstration

**Figure 6:**
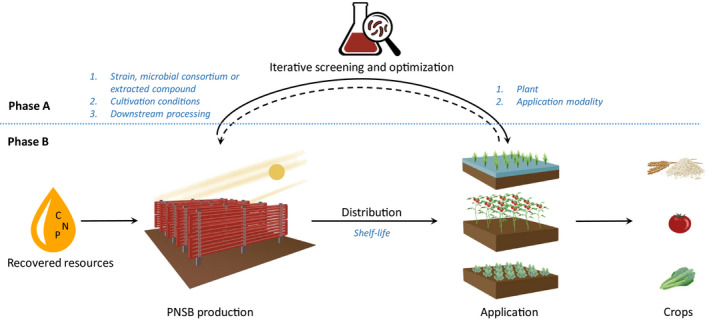
Roadmap for research (phase A) and valorization (phase B) of purple non‐sulphur bacteria products used for plant production. The iterative process of selecting a suitable product and application modality for each plant cultivation is visualized. Key parameters concerning each process are presented in italics. Depicted application concerns the most commonly used plant types, namely rice, tomato and pak choi, based on existing literature (Tables 1 and 2).

The research gaps arisen through reviewing the existing literature can be divided in two categories: (i) PNSB production and (ii) application (Fig. 6). Regarding production, parameters to be taken into account include the selection of a suitable PNSB product (strain, microbial consortium or extracted compound), the cultivation conditions and the downstream processing. Given the generic nature of the latter two, they will not be elaborated in the present review. Concerning the application, two parameters will be discussed, namely (i) plant selection and (ii) application modalities. Even though plant selection will not be further elaborated, it should be noted that more trials are required in order to establish the plant types for which PNSB products are suitable. As can be seen in Fig. 6, the selection of a suitable product for each application is an iterative process, requiring a strong link between research (phase A) and valorization (phase B).

#### PNSB strain selection

Choosing a strain for a specific application is not straightforward, as many criteria need to be met. For example, Maudinas *et al*. (1981) inoculated *R. capsulatus* B10 in a hydroponic medium (deprived of a nitrogen source), and this strain alone was unable to sustain plant growth in such a system. On the other hand, Elbadry and Elbanna (1999) used *R. capsulatus* DSM 155 in a similar experiment, where normal plant growth was observed. Parameters to be considered during strain selection include the ability to grow under micro‐aerobic conditions (Kantha *et al*., 2015), the potential to produce IAA and ALA, to fix nitrogen, and to produce soluble phosphate (Koh and Song, 2007; Lee *et al*., 2008). Furthermore, the carbon assimilation profiles give an indication about the spectrum of plant metabolites that can be used from each microbe, indicating the suitability as plant growth‐promoting rhizobacteria (Wong *et al*., 2014). Depending on the envisaged application, the extent of limiting the adverse effects of abiotic stresses plays an important role, as well as the HM bioremediation potential (Sakpirom *et al*., 2017). The isolation from environments with conditions similar to the envisaged application seems to be a promising approach (Fan *et al*., 2012; Sakpirom *et al*., 2017). In the study of Sakpirom *et al*. (2017), only seven from 235 PNSB isolates showed fertilizing potential based on their ${\text{NH}}_{4}^{+}$ release, whereas only four strains were able to remove HM and reduce CH_4_ emissions, illustrating the complexity of finding promising strains. The authors of this study stated that ‘*in order to obtain promising strains for field applications, both extensive and intensive efforts are required*’ (Sakpirom *et al*., 2017). This is in agreement with the findings of Lo *et al*. (2018), who performed a whole‐genome sequencing and analysis of *R. palustris* strains PS3 and YSC3 isolated on paddy soils. Even though both strains contained genes associated to plant growth‐promoting functions, the strain YSC3 did not enhance plant growth, which led to the conclusion that responses towards and interactions with plant hosts are essential. Furthermore, given that the combination of strains leads to better results than individual strains (Batool *et al*., 2017), research should focus on the use of synthetic communities to achieve the highest potential efficiency. For instance, the combination of *R. capsulatus* with *A. vinelandii* (Maudinas *et al*., 1981) as well as *R. palustris* CS2 with *R. faecalis* (Batool *et al*., 2017) resulted in better fertilization performance than application of individual strains. Mixed cultures outperform monocultures for a combination of diverse abilities, such as plant growth promotion, bioremediation of HM and GHG mitigation.

#### The unknown potential of PNSB‐derived products

The limited amount of studies and parameters tested concerning the use of cultivation supernatants or extracted PGPS does not enable the complete evaluation of this product (Appendix S1 in section 2). Even though the application of ALA‐containing PNSB cultivation supernatant restored the rice plant growth to the level of unfertilized plants not exposed to salinity stress, it resulted in lower growth performance than comparable dosage of commercially available ALA, indicating the presence of growth‐inhibiting compounds (Nunkaew *et al*., 2014a). Apart from the inhibiting effect of these compounds when applied in their biomass‐free form during batch tests, there is no evidence of their effect when accumulated in the field. Consequently, further research is required to establish the type of these inhibiting compounds and the mechanism of their production, as well as to elucidate their mechanism of action.

#### Application modalities

This literature review revealed that the required frequency and dosage of application remain yet undefined. For instance, the use of dried *R. sphaeroides* NR3 on spinach increased the yield (68%) when supplying 1.6 g dry PNSB cells, while the lower dosages did not have any effect (Kondo *et al*., 2008). On the other hand, the yield of mustard spinach increased (7.0%) only at the lowest dosage (0.28 g dry PNSB) (Kondo *et al*., 2008). Furthermore, Kondo *et al*. (2010) reported that the amount and times of application had no effect on fresh weight, Brix sugar content, titrable acidity, carotenoid and citric acid content, while they affected ascorbic, malic and phosphoric acid content of tomato fruit. Regarding the use of living cells, results show that weekly application of living PNSB on soil is sufficient to maintain the microbial populations for four (Lee *et al*., 2008) to 8 weeks (Wong *et al*., 2014) or for at least 17 days in a hydroponic system (Hsu *et al*., 2015), indicating that they can sustain their beneficial effects on plants. The large variation in frequency and dosage of application (Tables 1 and 2) did not enable making conclusions. Therefore, the effect of these parameters on plants, fruit and crops needs to be elucidated.

In addition, the reviewed studies did not present a clear pattern regarding the effect of each application method, due to the variability of the determined parameters. For example, Maudinas *et al*. (1981) inoculated *R. capsulatus* and *A. vinelandii* in a hydroponic nutrient solution (lacking a nitrogen source) for rice seedling growth (*Oryza sativa* L. cv. Delta). Normal growth, flowering and panicle formation were observed in 40% of the plants, indicating bacterial N_2_ fixation. Nevertheless, *R. capsulatus* alone was unable to sustain plant growth in such a system, as indicated by the signs of nitrogen deficiency in all plants and the fact that only 10% of the plants had formed panicles at the time of harvesting. On the other hand, Elbadry and Elbanna (1999) noted normal plant growth in similar experiments. Another example concerns the use of carrier material. When Harada *et al*. (2005) inoculated *R. palustris* KN122 at the floodwater of rice plants (*Oryza sativa* L. cv. Nipponbare), with and without the addition of rice straw, the shoot dry weight was not affected by the treatments. It was also observed that the total number of tillers and number of productive tillers was not affected by the inoculation when straw was not used, while they increased by 10–30% and 15% resp. when straw was supplied (Harada *et al*., 2005).

Furthermore, foliar spray of PNSB cells is a promising method to enhance photosynthesis as well as increase the glucoside content of stevia plants (Wu *et al*., 2013). However, the most significant effect was observed by the combination of foliar spray and irrigation, rather than each method individually, due to the combination of plant growth‐promoting effects of the two different application modes (Wu *et al*., 2013). Consequently, further research should be performed to establish the optimal fertilization strategy. Furthermore, foliar fertilizers containing PNSB are reported to contribute to disease resistance through the successful colonization of the phyllosphere (Su *et al*., 2017). Even though disease suppression through the use of microbes is theoretically an attractive solution, in reality, the alteration of environmental conditions due to the presence of viruses hinders the application potential (Atehnkeng *et al*., 2016; Cray *et al*., 2016). Given that studies using PNSB are scarce, no conclusion could be drawn about the optimal application method to promote disease resistance. Furthermore, no data were found regarding the effect of using PNSB products on microbial or parasitic plant diseases. Therefore, studies should focus on the effect of different PNSB application methods on the suppression of plant diseases.

The reviewed literature did not present a clear pattern regarding the effect of PNSB use on plant or/and crop pigmentation. For instance, the use of dried PNSB yielded 23–54% higher chlorophyll content in mustard spinach (Kondo *et al*., 2004, 2008), while similar treatments had no positive effect on the chlorophyll content of spinach (Kondo *et al*., 2004). Additionally, the use of live PNSB increased the chlorophyll content of stevia by 30–88% (Wu *et al*., 2013), whereas similar treatment did not present a significant effect on Chinese dwarf cherry (4.8% increase) (Yin *et al*., 2012). In addition, there are no available data regarding the long‐term effect of the use of PNSB in HM‐contaminated fields, as well as whether the HM content in the crop is reduced through the treatment. Finally, as highlighted by Pikaar *et al*. (2018), elaborated field trials are required in order to establish whether the use of microbial products can increase the SOC of agricultural soils.

### From research to implementation: Reflections on shelf life and application methods

To our knowledge, currently no PNSB products are available on the global market. Whereas the parameters to be considered during the production of bacterial inoculants have been summarized in the past (Bashan *et al*., 2014), an important remark concerning the industrialization of PNSB products concerns the costs of their production. Given their importance, the economic aspects are discussed separately (section A preliminary cost effectiveness analysis on PNSB).

A key concern at the distribution level is the shelf life of living inocula. Even though there are no available data regarding PNSB products, there is evidence that the shelf life of these liquid cultures is longer than 2 years at temperatures below 20°C (Catroux *et al*., 2001), and they can even tolerate temperatures up to 55°C (Mahdi *et al*., 2010). Future studies could focus on optimizing preservation conditions, through slowing down decay rates.

Finally, several application methods suggested in literature (Appendix S1 in section 1) are suitable for small‐scale cultivation due to their labour‐intensive nature, which can be translated to increased costs. For instance, seedling dipping in PNSB products requires a considerable amount of effort, whereas soil application, foliar spraying and seed coating seems to be possible on a large‐scale due to the similar equipment already available. Therefore, further investigations are required in order to target potential consumers of each product type.

### A preliminary cost effectiveness analysis on PNSB

A preliminary cost effectiveness analysis was performed by comparing the price (cost and profit margin) for delivering the different functionalities of PNSB with the current market price of products with comparable properties. Parameters contributing to the production costs include the cultivation medium and the need to provide sterile conditions. Concerning the former, as explained in section Sustainability of PNSB production, PNSB can be produced on recovered resources such as wastewaters (Verstraete *et al*., 2016). Therefore, they can contribute to resource recovery through the immobilization of nutrients from anthropogenic sources (i.e. wastewaters) (Hülsen *et al*., 2014; Alloul *et al*., 2019), while eliminating the need for external nutrient supply. Given that PNSB use the infra‐red light spectrum, coating the reactors with a membrane permitting the penetration of this light spectrum would facilitate selective growth (Alloul *et al*., 2019), however, increasing the investment costs. Nevertheless, provided that selective production in raceway ponds can be established, this would lower the overall costs. Given the forthcoming related innovations, these two parameters are not considered in the present cost analysis.

To our knowledge, only, Alloul *et al*. (2019) reported estimated production costs for photoheterotrophic PNSB production under European conditions. This economic estimation was based on a closed photobioreactor fed with recovered resources (brewery effluent) and included harvesting and downstream processing with ultrafiltration, centrifugation and spray drying as to obtain a biomass powder (i.e. dead cells). A production cost of €10.0 kg_DW_^−1^ was estimated, which is comparable to the cost of producing dried microalgae (€12.6 kg_DW_^−1^) (Acién *et al*., 2012), while avoiding a wastewater treatment cost for the removed pollutants. Production of living PNSB cells can cut the drying cost and can be estimated at €8.94 kg_DW_^−1^ (details about the calculations can be found in Appendix S1 in section 5.1). Furthermore, innovations in PNSB cultivation methods, such as the use of raceway ponds, may further lower costs as would production in low‐income regions. To estimate a market price, the costs for packaging and logistics should be added, along with a profit margin. Adding an arbitrary estimate of 30%, prices could be around €11.6 – €13.0 kg_DW_^−1^ for wet – dried PNSB biomass.

The current rough price estimates show that PNSB products are economically promising. Specifically, the current price of organic N and P fertilizers is €62 kg_N_^−1^ and €63 kg_P_^−1^, which is one order of magnitude lower than the costs estimated based on the study of Alloul *et al*. (2019), namely €153 kg_N_^−1^ and €542 kg_P_^−1^ derived from PNSB (Appendix S1 in section 5.2). Using the considerations discussed in section Resistance to stresses, prices between €446–35 692 kg_IAA_^−1^ and €2937–100 870 kg_ALA_^−1^ are estimated. This is comparable with the commercial prices found in the ranges of €13–261 kg_IAA_^−1^ and €1740–8700 kg_ALA_^−1^ (Appendix S1 in section 5.3). The prior analysis compared all compounds separately, while the production of PNSB can yield the joint value of these, lowering the actual price of each individual product (i.e. PNSB biomass, IAA and ALA are produced concurrently).

From an economic perspective, the probiotic effects of PNSB may be more appealing, yet they are also extremely challenging to assess in monetary terms. When viable PNSB cells are probiotically active, they can combine effects of N fixation, P release, increased stress resistance and bioremediation, amongst others. Furthermore, such application only requires small amounts of PNSB to serve as inoculum (Tables 1 and 2), pulling the focus more to the appropriate management of soil microbiota. For rice paddy fields, evidence already showed that common practice (e.g. use of straw) can enhance the growth of PNSB at no additional costs (Kantha *et al*., 2015; Kantachote *et al*., 2016); and as a result, multiple benefits of N fixation, HM remediation and CH_4_ emission reduction can be harnessed. In conclusion, it should be kept in mind that commercial PNSB production is rather new, and that it is likely that further technological innovation and associated efficiency gains will decrease its price.

## Conclusions

Purple non‐sulphur bacteria present promising results in plant cultivation as they combine multiple functions: direct and indirect fertilization, biostimulation and biofortification as well as demonstrating environmental benefits. Paddy fields provide favourable conditions for these photosynthetic bacteria to grow (photoheterotrophically, micro‐aerobically), therefore, allowing them to unfold their full potential in enhancing rice plant growth, harvest yield and quality, reinforcing the resistance to environmental stresses while reducing environmental footprint of rice production. However, the synergies involved are not yet fully understood. Further research is required to establish the optimal strain (or microbial consortium), frequency, formulation and dosage of each application and evaluate the effect of the PNSB metabolites on plant growth and environmental parameters. Answering these questions should pave the way for the use of PNSB products in a variety of agricultural applications leading to a better and more sustainable food production.

## Conflict of interest

None declared.

## Supporting information


**Appendix S1**. Details regarding the application methods of PNSB, effects of PNSB on plant cultivation, and economic aspects of PNSB production addressed in the review.
