# Supplementary material for: Purple non‐sulphur bacteria and plant production: benefits for fertilization, stress resistance and the environment
Source: Microb Biotechnol. 2019 Aug 21;13(5):1336–65. doi: 10.1111/1751-7915.13474 (PMC7415370; doi:10.1111/1751-7915.13474)
Supplement: Supplementary file 1 — Appendix S1. Details regarding the application methods of PNSB, effects of PNSB on plant cultivation, and economic aspects of PNSB production addressed in the review. [file MBT2-13-1336-s001.docx]

**SUPPORTING INFORMATION**

**Purple non-sulphur bacteria and plant production: Benefits for fertilization, stress resistance and the environment**

Myrsini Sakarika^1^, Janne Spanoghe^1^, Yixing Sui^1^, Eva Wambacq^2^, Oliver Grunert^3^, Geert Haesaert^2^, Marc Spiller^1^, Siegfried E. Vlaeminck^1*^

^1^Research Group of Sustainable Air, Energy and Water Technology, Department of Bioscience Engineering, University of Antwerp, Groenenborgerlaan 171, 2020 Antwerpen, Belgium

^2^Department of Plants and Crops, Faculty of Bioscience Engineering, Ghent University, V. Vaerwyckweg 1, 9000 Ghent, Belgium

^3^Greenyard Horticulture Belgium NV, Skaldenstraat 7a, 9042 Gent, Belgium

^*^Corresponding author: [siegfried.vlaeminck@uantwerpen.be](mailto:siegfried.vlaeminck@uantwerpen.be)

Number of pages: 23

Number of Tables: 10

Contents

[1. Application methods of PNSB products 3](#_Toc14775027)

[2. Literature concerning extracted compounds 3](#_Toc14775028)

[2.1 Cultivation supernatant 3](#_Toc14775029)

[2.2 Rhodestrin and rhodethrin 3](#_Toc14775030)

[2.3 Provision of extracted compounds 3](#_Toc14775031)

[2.3.1 Plant performance 5](#_Toc14775032)

[2.3.2 Resistance to abiotic stresses 5](#_Toc14775033)

[3. Evaluating use of PNSB as fertilizer, bio-stimulant and bio-fortifier 7](#_Toc14775034)

[3.1 Overall plant growth performance 7](#_Toc14775035)

[3.2 Yield and quality increase of edible plant biomass 9](#_Toc14775036)

[4. Relative effect of PNSB products on plants 9](#_Toc14775037)

[4.1 Effect of dead cells 10](#_Toc14775038)

[4.2 Effect of living cells 13](#_Toc14775039)

[4.3 Effect of PNSB extracted substances 18](#_Toc14775040)

[5. Economic calculations 19](#_Toc14775041)

[5.1 PNSB biomass production costs 19](#_Toc14775042)

[5.2 Price based on nitrogen and phosphorus content 19](#_Toc14775043)

[5.3 Indole-3-acetic acid (IAA) and 5-aminolevulinic acid (ALA) production from PNSB 20](#_Toc14775044)

[6. References 21](#_Toc14775045)

# Application methods of PNSB products

PNSB products with dead cells contain either autoclaved cells or dried biomass produced through freeze-drying, spray-drying or heat-drying. These cells can be applied in soil directly, where nutrients can be transferred from the root to the rest of the plant.

Living PNSB cells can be applied via the soil by either direct application or through irrigation with suspension containing the cells. This application method permits the use of carriers such as compost, peat or straw, which facilitate the attachment of the non-native microbes provided (Kantha *et al.*, 2015; Kantachote *et al.*, 2016). Additionally, plant seeds can be coated with living PNSB biomass, providing a direct contact of PGPS produced by the living cells to the plant at an early growth stage (Elbadry and Elbanna, 1999; Gamal-Eldin and Elbanna, 2011; Rana *et al.*, 2016; Batool *et al.*, 2017). Another similar practice is to dip the seedling roots into a living PNSB biomass suspension for about 30 minutes (Elbadry *et al.*, 1999). The microbes attach on the seedling roots and can therefore promote the plant’s growth while increasing their numbers. In this case, a single application of a relatively small amount of fertilizer is performed, while the number of living cells will increase as the plant grows (Gamal-Eldin and Elbanna, 2011). In a hydroponic cultivation system, living PNSB cells can be inoculated directly into the liquid cultivation medium (Maudinas *et al.*, 1981). As PNSB grow faster in a liquid medium than in a solid medium, a higher quantity of PGPS can be produced promoting better plant growth. Besides the root fertilization, foliar fertilization can also be applied with liquid fertilizer containing living cells (Fageria *et al.*, 2009). During foliar fertilization, plants can absorb nutrients through their leaves, and the absorption takes place through their stomata and epidermis. With this fertilization method, the leaching out of nutrients in the soil is avoided. Nevertheless, it requires a large leaf area for sufficient nutrient absorption (Fageria *et al.*, 2009). Regardless of the application method, multiplication of the inoculated cells should occur to avoid out-competition by the native soil microbes, allowing the foliar fertilizer to be beneficial (Chen, 2006).

# Literature concerning extracted compounds

## Cultivation supernatant

The use of the cultivation supernatant as liquid fertilizer containing extracted PGPS is a rather straightforward process, similar to the supplementation of conventional liquid fertilizers. One possible way of supplying PGPS to the plant is through dipping the seedling roots into the culture supernatant where the absorbed PGPS can unravel their promoting effects during the plant growth (Nunkaew *et al.*, 2014). Another option is to apply it on the plant aerial tissues (e.g. foliar spraying) (Cabello-Conejo *et al.*, 2014).

## Rhodestrin and rhodethrin

Two novel PGPS were isolated from the culture supernatants of *Rhodobacter sphaeroides*: rhodestrin and rhodethrin. The compounds were identified as indole terpenoid esters and both tested positive in an auxin bioassay with coleoptiles (Sunayana *et al.*, 2005; Ranjith *et al.*, 2007).

## Provision of extracted compounds

Table 1 presents the literature concerning the extracted PGPS.

***Table 1:*** *Overview of literature concerning the use of extracted plant growth promoting substances (PGPS) derived from PNSB. All strains were cultivated under photoheterotrophic conditions unless stated otherwise. Details about the application method and fertilization effect are presented.*

| **PNSB** | **Microbial fertilizer** | | | **Plant** | | **Reference** |
| --- | --- | --- | --- | --- | --- | --- |
| **Strain** | **Compound** | **Composition and dosage** | **Application method** | **Species** | **Effect** |  |
| *Rhodobacter sphaeroides* OU5 | extracted rhodestrin | 50 nM of rhodestrin in Murashige and Skoog medium; once | coleoptile assay | *Morus alba* | promoted root initiation; faster rooting initiation; higher root-to-shoot ratio | Sunayana *et al.* (2005) |
| *Rhodobacter sphaeroides* OU5 | extracted rhodethrin | 0.5 µM of rhodetrin in MS (Murashige and Skoog) medium; once | coleoptile assay | *Morus alba* | promoted root initiation; induced rooting and shooting; faster root initiation; increased root length | Ranjith *et al.* (2007) |
| *Rhodopseudomonas palustris* TN114 and PP803 | ALA containing supernatants | 10x-diluted PNSB culture supernatant (with 2.57 μM ALA for TN114; 2.11 μM for PP803) without and with salt stress; once for 12h | seedlings | *Oryza sativa* L. | without and with salt stress: increased dry and fresh weight; increased root length; increased chlorophyll content;  with salt stress: protective effect towards salinity stress (prevented inhibitions) | Nunkaew *et al.* (2014) |

### Plant performance

Rhodestrin and rhodethrin, two indole terpenoid phytohormones were extracted from *Rhodobacter sphaeroides* OU5 (Sunayana *et al.*, 2005; Ranjith *et al.*, 2007), and were used in tissue cultures of mulberry (*Morus alba*). 0.05 μM rhodestrin was compared to 10x more concentrated (0.5 μM) commercially available auxins (i.e. naphthalene acetic acid (NAA) and 2,4 dichloro-phenoxy acetic acid (2,4-D)). Rhodestrin induced rooting and increased the root-to-shoot ratio, while rooting was induced in half the time (6–8 days) compared to the other auxins (12–15 days) (Sunayana *et al.*, 2005). Similar results were obtained with 0.5 μM rhodethrin, which resulted in better plant performance than 5 μM IAA, in terms of root length (data not available) as well as root initiation time (8–10 days in contrast to 12–15 days) (Ranjith *et al.*, 2007). The authors of these studies commented that since many indole terpenoid esters are commonly produced through chemical reactions, their biological production from PNSB is worth exploiting (Sunayana *et al.*, 2005; Ranjith *et al.*, 2007). Nunkaew *et al*. 2014 used 10x diluted *Rhodopseudomonas palustris* TN114 and PP803 cultivation supernatant containing 2.7 and 2.1 μM ALA, respectively on rice seedlings (*Oriza sativa* L.). *R. palustris* TN114 supernatant presented comparable results to 1 μM commercial ALA. Specifically, the seedling fresh and dry weight increased by 18% and 78% with TN114 supernatant while PP803 supernatant did not have notable effects. The root length increased by 158% and 137% from TN114 and PP803 supernatant respectively. Finally, total chlorophyll content rose by 13% and 16% from TN114 and PP803 supernatant respectively.

### Resistance to abiotic stresses

The effect of 10x diluted cultivation supernatant of ALA-producing *Rhodopseudomonas palustris* PP803 and TN114 were compared to the use of commercial ALA, in rice plants (*Oriza sativa* L.) cultivated under salinity stress (Nunkaew *et al.*, 2014). The PNSB supernatant resulted in increased plant dry (98 and 120% for strains PP803 and TN114 respectively) and wet weight (84% and 72% respectively). The addition of TN114 supernatant improved the relative root growth (%RRG) comparably to the commercial ALA (178 %RRG). Increased chlorophyll content was observed in all cases (475, 503 and 496% for commercial ALA, 10X diluted TN114 or PP803 supernatant, respectively). All treatments promoted the reduction of damaging ROS such as H_2_O_2_ with the best effect observed under the commercial ALA treatment.

***Table 2:*** *Overview of literature concerning the use of extracted plant growth promoting substances (PGPS) derived from PNSB. All strains were cultivated under photoheterotrophic conditions unless stated otherwise. Details about the application method and fertilization effect are presented.*

| **PNSB** | **Microbial fertilizer** | | | **Plant** | | **Reference** |
| --- | --- | --- | --- | --- | --- | --- |
| **Strain** | **Compound** | **Composition and dosage** | **Application method** | **Species** | **Effect** |  |
| *Rhodobacter sphaeroides* OU5 | extracted rhodestrin | 50 nM of rhodestrin in Murashige and Skoog medium; once | coleoptile assay | *Morus alba* | promoted root initiation; faster rooting initiation; higher root-to-shoot ratio | Sunayana *et al.* (2005) |
| *Rhodobacter sphaeroides* OU5 | extracted rhodethrin | 0.5 µM of rhodetrin in MS (Murashige and Skoog) medium; once | coleoptile assay | *Morus alba* | promoted root initiation; induced rooting and shooting; faster root initiation; increased root length | Ranjith *et al.* (2007) |
| *Rhodopseudomonas palustris* TN114 and PP803 | ALA containing supernatants | 10x-diluted PNSB culture supernatant (with 2.57 μM ALA for TN114; 2.11 μM for PP803) without and with salt stress; once for 12h | seedlings | *Oryza sativa* L. | without and with salt stress: increased dry and fresh weight; increased root length; increased chlorophyll content;  with salt stress: protective effect towards salinity stress (prevented inhibitions) | Nunkaew *et al.* (2014) |

# Evaluating use of PNSB as fertilizer, bio-stimulant and bio-fortifier

## Overall plant growth performance

The use of dead PNSB cells has shown the enhancement of shoot growth in a variety of plants. Specifically, when Kondo *et al.* (2004) provided freeze-dried *R. sphaeroides* on mustard spinach (*Brassica campestris*) cultivated under different light qualities, the shoot fresh weight and length were up to 17% and 9% higher, respectively. Similarly, Koh and Soh (2007) used autoclaved cells of *Rhodopseudomonas* sp. BL6 and KL9 on tomato plants (*Solanum lycopersicon* Mill. cv. Poongyoung) and observed an increase of resp. 98% and 49% on the plant’s dry weight, nevertheless, the effect on shoot length was negligible. Kondo *et al*. (2008) applied PNSB powder on spinach (*Spinacia oleracea*). The application of dry PNSB had positive effects on shoot fresh weight, dry weight and length of spinach (resp. 68%, 59% and 26% increase). When similar experiments were performed on mustard spinach (*Brassica campestris*), the PNSB biomass addition increased both fresh and dry shoot weight (7% and 23% increase resp.), while it did not have positive effects on the shoot length (Kondo *et al.*, 2008). The addition of autoclaved *Rhodopseudomonas* sp. KL9 cells increased the shoot dry weight (34%) of tomato plants (*Solanum lycopersicon* Mill. cv. Zeus) (Lee *et al.*, 2008). However, when Wong *et al*. (2014) fertilized pak choi (*Brassica rapa* ssp. *chinensis*) with heat-killed *R. palustris* PS3, YSC3 and YSC4, no significant effect on growth of was noted (Wong *et al*., 2014). Similarly, the use of foliar spray containing autoclaved *R. palustris* GJ-22 on tobacco plants (*Nicotiana tabacum* L. cv. Samsun NN) did not exhibit a significant effect on plant growth parameters (Su *et al.*, 2017).

The use of living cells has been more extensively explored (Figure 2; Tables 1-2 in manuscript) and the results are promising. When Koh and Soh (2007) inoculated tomato seeds (*Solanum lycopersicon* Mill. cv. Poongyoung) with *Rhodopseudomonas* sp. BL6 and KL9, the plant was 37% and 71% longer from BL6 and KL9 resp., while the dry weight of the seedlings increased resp. by 174% and 264%. Similar results were obtained by Lee *et al.* (2008) in tomato plants (*Solanum lycopersicon* Mill. cv. Zeus), where soil irrigation with *Rhodopseudomonas* sp. BL6 and KL9 suspensions increased shoot dry weight (resp. by 47% and 121%) and shoot length (resp. by 18% and 35%). Apart from the elongation of the shoot, the shoot diameter also enlarged. Yin *et al.* (2012) used a foliar spray containing *R. palustris* suspension on Chinese dwarf cherry seedlings (*Prunus humilis* Bunge), where the fresh weight of leaves and the leaf area increased by 44% and 37% respectively. When *Rhodopseudomonas* sp. (ISP-1) was provided to stevia plants (*Stevia rebaudiana*), the shoot fresh weight increased by 26–68% (Wu *et al.*, 2013). Wong *et al*. (2014) added *R. palustris* PS3, YSC3 and YSC4 at 50% of the standard fertilizer dosage for pak choi (*Brassica rapa* ssp. *chinensis)*. All strains enhanced the fresh and dry shoot weight on plants grown from old (stored) seeds, with the best results presented by strain PS3 (44% and 67% resp.). Moreover, Batool *et al*. (2017) supplied *R. palustris* CS2 and *R. faecalis* SS5 on bean seeds (*Vigna mungo*), where an increase in the fresh and dry seedling weight (14–21% and 0–50%) as well as in shoot length (17–22%) was noted. The combination of the two strains resulted in higher growth enhancement, amounting up to 15% more elongated shoots. Finally, the foliar fertilization of tobacco plants (*Nicotiana tabacum* L. cv. Samsun NN) with *R. palustris* GJ-22 resulted in 55% taller plants (Su *et al.*, 2017). The strain successfully colonized the phyllosphere, increasing tobacco yield by 31%, while the number of first-class leaves increased by 34%. Even though the majority of studies report positive effects of PNSB inoculation on plant performance, Xu *et al*. (2018) did not observe any significant enhancement in the growth of stevia (*Stevia rebaudiana*) during field trials with *R. palustris*.

Most studies using living PNSB investigated the effect on rice plants (Table 2 in manuscript). Specifically, Elbadry and Elbanna (1999) cultivated rice seedlings (*Oryza sativa* L. cv. Giza 159, Giza 171, Giza 176 and Giza 181)) in a hydroponic nutrient solution inoculated with *R. capsulatus* DSM 155, without and with combined nitrogen source. The shoot was taller in all cases, with the biggest effect presented in the case of no nitrogen addition (52–75%) compared to nitrogen supplementation (1.9–8.3%). The shoot dry weight significantly increased (47–100%) when nitrogen was not present in the medium. Furthermore, Elbadry *et al*. (1999) inoculated rice seedling roots (*Oryza sativa* L. Giza 176) with *R. capsulatus* DSM 155 cell suspension. The treatment resulted in a 24% increase of shoot dry weight, whereas the shoot height was not significantly affected (1.9% increase). The straw yield increased by 19%, while panicle length and weight increased by 6.5% and 2.2% resp. (Elbadry *et al.*, 1999). When Gamal-Eldin and Elbanna (2011) coated rice seeds (*Oriza sativa* L. cv. Giza 177) with *R. capsulatus* DSM 155, the shoot height and weight was 6.9% and 19% higher respectively. The inoculation of a mixed carrier (containing rice straw and husk ash) with *R. palustris* was tested in organic as well as saline conditions (Kantha *et al.*, 2015; Kantachote *et al.*, 2016). *R. palustris* TK103, PP803, and P1 increased the shoot dry weight and length (48–77% and 19–23% resp.) of rice (*Oryza sativa L*. ssp. indica cv. Pathumthani) under normal conditions (Kantha *et al.*, 2015). On the other hand, Kantachote et al. (2016) did not observe any effect on the maximum height of rice plants (cv. KDML 105), while the flowering and harvesting days were prolonged by 2.5–7.4% and 1.7–5.2% resp. when using the stains TN114, PP803 and TK103. Nevertheless, the panicle height and weight increased in all cases (5.9–8.8% to 14–24% resp.). The effect of PNSB was more significant under salt stress (0.25% NaCl), and the best results were exhibited by *R. palustris* PP803 (Kantha *et al.*, 2015; Kantachote *et al.*, 2016).

It has been demonstrated that the use of dead or living PNSB affects the growth of the roots. Specifically, the fresh and dry weight of mustard spinach (*Brassica campestris*) root increased resp. by 61%, 150% under the blue light treatment through the use of dry PNSB (Kondo *et al.*, 2004), and by 136% and 125% resp. under normal light conditions. Furthermore, autoclaved cells of *Rhodopseudomonas* sp. KL9 increased the root dry weight of tomato plants (*Solanum lycopersicon* Mill. cv. Zeus) by 26% (Lee *et al.*, 2008). On the other hand, the use of the living strain resulted in 79% increase of root dry weight (Lee *et al.*, 2008). Tobacco plant (*Nicotiana tabacum* L. cv. Samsun NN) roots were also benefitted by the fertilization with living *R. palustris* GJ-22 as demonstrated by the enhanced seed germination rate (21%) and root length (50%) (Su *et al.*, 2017). The use of *R. palustris* CS2 and *R. faecalis* SS5 on bean plants (*Vigna mungo*) increased the root length by 13–19% while the combination of the two strains resulted in 24% more elongated roots (Batool *et al.*, 2017). Rice plant roots (*Oryza sativa L*. ssp. indica cv. Pathumthani) are also benefited by the inoculation of living *R. palustris* TK103, PP803, and P1 as indicated by the increase in root dry weight and length (73–155% and 8.3–11% resp.) (Kantha *et al.*, 2015). However, Elbadry and Elbanna (1999) reported that the roots of inoculated with *R. capsulatus* DSM 155 rice plants (*Oryza sativa* L. cv. Giza 159, Giza 171, Giza 176 and Giza 181)) were shorter compared to the negative control (-9.4 up to -37%), but the number of lateral roots was higher and hence the root dry weight increased (2.1–9.8%). The latter is supported by Duca *et al*. (2014) who observed that bacterial IAA stimulates the root hair formation.

## Yield and quality increase of edible plant biomass

The use of PNSB enhances both quantity and quality of harvested crops. More specifically, the use of *R. capsulatus* powder on rice resulted in 32% improved yield (Kobayashi and Haque, 1971), while dried *R. sphaeroides* NR3 increased the yield of spinach by 68% and the yield of mustard spinach by 7.0% (Kondo *et al.*, 2008). Furthermore, autoclaved *Rhodopseudomonas* sp. BL6 and KL9 cells significantly promoted the average tomato fruit yield (42–50% increase) while inoculation with the same amount of living BL6 and KL9 enhanced the yield by 21–98% (Lee *et al.*, 2008); and mandarin fruit yield increased (27%) through the use of living PNSB cells (Kobayashi and Tchan, 1973). Inoculation with *R. capsulatus* increased the rice yield by 20% (Yoshida *et al.*, 1991); living *R. palustris* improved the pak choi yield up to 79% (Xu *et al.*, 2016); while foliar spray containing mixed PNSB cultures resulted in grape yield increase of 6.9% (Shi *et al.*, 1995). Furthermore, Elbadry *et al*. (1999) demonstrated that the inoculation with *R. capsulatus* DSM 155 increased the rice yield by 30%. According to this study, the application of the recommended nitrogen fertilization rate (95 kg N ha^-1^) in combination with inoculation with PNSB can lead to an increase of 1.2 ton rice grain yield ha^-1^ (Elbadry *et al.*, 1999). Gamal-Eldin and Elbanna (2011), observed a significantly enhanced grain yield (14–30% or 1.7–2.1 t ha^-1^) when *R. capsulatus* was inoculated in paddy fields, especially in saline conditions (18–33% compared to 11–27% in non-saline conditions). The most prominent effect was presented when no nitrogen source was supplied (average increase of 30%), however, the net yields were always higher in non-saline field conditions (Gamal-Eldin and Elbanna, 2011). Finally, inoculation with several *R. palustris* strains (TN114, PP803, TK103) improved rice grain yield by up to 19% (Kantachote *et al.*, 2016).

# Relative effect of PNSB products on plants

Tables 2-4 present the results concerning dead cells. Specifically, Table 2 present the effect on plant performance, Table 3 presents the effects on the crop (edible plant biomass) and Table 4 presents the effects on the pigments of the leafy vegetables and crops. The same results are presented in Tables 5-7 for living PNSB products. Finally, Table 8 presents the effects on extracted PGPS (from PNSB cultivation supernatant).

## 4.1 Effect of dead cells

Table 3: Effect of dead PNSB supplementation on plant performance. Relative change in characteristics compared to the control. Numbers are presented in percentages (%).

| PNSB strain | Dosage | Type of plant | Control | Effect (% of control) | | | | | | | Reference |
| --- | --- | --- | --- | --- | --- | --- | --- | --- | --- | --- | --- |
|  |  |  |  | Seed germination rate | Shoot fresh weight | Shoot dry weight | Shoot length | Root fresh weight | Root dry weight | Total Chlorophyll content |  |
| *Rhodobacter sphaeroides* | N.A. | mustard spinach | no PNSB powder addition | - | -4.8 | 17.5 | -2.9 | 61.1 | 150.0 | 54.1 | Kondo *et al.* (2004) |
|  |  |  |  | - | 0.7 | -14.3 | 5.5 | -4.2 | -28.6 | 0.0 |  |
|  |  |  |  | - | 16.5 | -1.4 | 8.9 | 0.0 | -16.2 | 0.0 |  |
| *Rhodopseudomonas* sp. BL6 | 5 ∙ 10^7^ autoclaved cells^1^ | tomato | sterile water | -3.3^2^ | - | 98.4^3^ | -3.8 | - | - | - | Koh and Song (2007) |
| *Rhodopseudomonas* sp. KL9 | 5 ∙ 10^7^ autoclaved cells^1^ |  |  | 3.8^2^ | - | 49.2^3^ | 8.3 | - | - | - |  |
| *Rhodopseudomonas* sp. BL6 | 2.24 ∙ 10^11^ autoclaved cells^4^ | tomato | sterile water | - | - | 2.8 | 0 | - | -2.4 | - | Lee *et al.* (2008) |
| *Rhodopseudomonas* sp. KL9 | 2.24 ∙ 10^11^ autoclaved cells^4^ |  |  | - | - | 33.6 | 0 | - | 26.2 | - |  |
| *Rhodobacter sphaeroides* NR3 | 0.4 g dried PNSB^5^ | spinach | no PNSB powder addition | - | -29.8 (21.2) | -31.1 (23.0) | -6.1 (8.5) | - | - | -32.8 (60.0) | Kondo *et al.* (2008) |
|  | 0.8 g dried PNSB^5^ |  |  | - | 0.0 (11.5) | -3.3 (0.0) | 9.5 (5.0) | - | - | -19.0 (48.9) |  |
|  | 1.6 g dried PNSB^5^ |  |  | - | 68.1 (30.8) | 59.0 (25.7) | 25.9 (27.0) | - | - | 0.0 (31.1) |  |
|  | 0.28 g dried PNSB^5^ | mustard spinach |  | - | 7.0 | 22.5 | -4.8 | 27.9 | 56.3 | 23.3 |  |
|  | 0.56 g dried PNSB^5^ |  |  | - | -1.0 | 11.0 | -2.1 | 35.6 | 68.8 | 35.0 |  |
|  | 1.12 g dried PNSB^5^ |  |  | - | -8.5 | 4.0 | -5.9 | 135.6 | 125.0 | 30.0 |  |
| *Rhodopseudomonas palustris* GJ-22 | N.A. (autoclaved cells)^6^ | tobacco^2^ | ultrapure water | 2.7 | 1.1 (3.1) | - | - | - | - | - | Su *et al.* (2017) |

Numbers in parenthesis indicate experiments with sterilized soil

^1^Seeds inoculated with 5 mL autoclaved bacterial suspension (10^7^ cells mL^-1^)

^2^Germination percentage

^3^Dry weight of whole plant

^4^4.0 ∙ 10^9^ cells per day (400 mL bacterial suspension at a concentration of 10^7^ cells mL^-1^)

^5^2.0∙ 10^9^ cells per week (10 mL bacterial suspension at a concentration of 2.0∙10^9^ MPN mL^-1^)

^6F^oliar spray to soaking wet of suspension with density of 6∙10^7^ CFU mL^-14^

Table 4: Effect of dead PNSB supplementation on edible plant biomass. Relative change in qualitative and quantitative characteristics compared to the control. Numbers are presented in percentages (%).

| PNSB strain | Dosage | Type of crop/ leafy vegetable | Control | Effect (% of control) | | | | | | | Reference |
| --- | --- | --- | --- | --- | --- | --- | --- | --- | --- | --- | --- |
|  |  |  |  | Fruit per flower | Number of crops per tree | Average fresh weight per crop | Total weight per harvest (yield) | Total water content | Brix sugar content | Titratable acidity |  |
| *Rhodopseudomonas capsulatus* | N.A. | rice | NH_4_Cl | - | - | - | 31.6 | - | - | - | Kobayashi and Haque (1971) |
| *Rhodobacter sphaeroides* | freeze-dried cells^1^ | mustard spinach | no PNSB powder addition | - | 2.3^4^ | -4.8 | - | - | - | - | Kondo *et al.* (2004) |
|  | freeze-dried cells^2^ |  |  |  | -2.0^4^ | 0.7 |  |  |  |  |  |
|  | freeze-dried cells^3^ |  |  |  | 2.0^4^ | 16.5 |  |  |  |  |  |
| *Rhodopseudomonas* sp. BL6 | 2.24 ∙ 10^11^ autoclaved cells^5^ | tomato | sterile water | -10.2 | - | - | 41.7 | - | - | - | Lee *et al.* (2008) |
| *Rhodopseudomonas* sp. KL9 | 2.24 ∙ 10^11^ autoclaved cells^5^ |  |  | 13.5 |  |  | 50.4 |  |  |  |  |
| *Rhodobacter sphaeroides* NR3 | 0.4 g dried PNSB^6^ | spinach | no PNSB powder addition | - | - | - | -29.8 (21.2) | -29.6 (20.9)^*^ | - | - | Kondo *et al.* (2008) |
|  | 0.8 g dried PNSB^6^ |  |  |  |  |  | 0.0 (11.5) | 0.5 (13.5)^*^ |  |  |  |
|  | 1.6 g dried PNSB^6^ |  |  |  |  |  | 68.1 (30.8) | 69.4 (31.6)^*^ |  |  |  |
|  | 0.28 g dried PNSB^6^ | mustard spinach |  | - | - | - | 7.0 | 5.5^*^ | - | - |  |
|  | 0.56 g dried PNSB^6^ |  |  |  |  |  | -1.0 | -2.1^*^ |  |  |  |
|  | 1.12 g dried PNSB^6^ |  |  |  |  |  | -8.5 | -9.7^*^ |  |  |  |
| *Rhodobacter sphaeroides* NR3 | 1.25 g dried PNSB^6^ | tomato | 1.25 g (NH_4_)_2_SO_4_ | - | - | 9.4 | - | - | -6.0 | -15.1 | Kondo *et al.* (2010) |
|  | 1.25 g dried PNSB^7^ |  |  |  |  | 9.0 |  |  | -3.4 | -15.8 |  |
|  | 2.5 g dried PNSB^6^ |  | 2.5 g (NH_4_)_2_SO_4_ |  |  | 7.8 |  |  | -3.6 | -9.6 |  |
|  | 2.5 g dried PNSB^7^ |  |  |  |  | -1.4 |  |  | -4.7 | -13.2 |  |

^*^calculated from graphs; numbers in parenthesis indicate experiments with sterilized soil

^1^irrigation (daily irrigation of 0.1 g/L PNSB cells dissolved in 10% Hoagland solution; quantity not specified): treatment with 100% blue light (470 nm); ^2^irrigation (daily irrigation of 0.1 g/L PNSB cells dissolved in 10% Hoagland solution; quantity not specified): treatment with 100% red light (660 nm); ^3^irrigation (daily irrigation of 0.1 g/L PNSB cells dissolved in 10% Hoagland solution; quantity not specified): treatment with 20% blue light (470 nm) - 80% red light (660 nm)

^4^Number of leaves per plant

^5^4.0 ∙ 10^9^ cells per day for 8 weeks (400 mL bacterial suspension at a concentration of 10^7^ cells mL^-1^)

^6^dosed once; ^7^split application over ten times

Table 5: Effect of dead PNSB supplementation on the content of pigments: chlorophyll a and b, individual carotenoid pigment (β-carotene and lycopene) and total carotenoid content content of crops and leafy vegetables, in comparison to the control (inorganic fertilizer). Numbers are presented in percentages (%).

| PNSB strain | Dosage | Type of crop/ leafy vegetable | Control | Chlorophyll a | Chlorophyll b | β-carotene | Lycopene | Total carotenoid | Reference |
| --- | --- | --- | --- | --- | --- | --- | --- | --- | --- |
| N.A. | N.A. | mandarin | 1L of 1:1000 diluted commercial liquid fertilizer (N: 10%; P: 5%; K: 5%) every 10 days | - | - | - | - | 5.8 | Kobayashi and Tchan (1973) |
| *Rhodobacter sphaeroides* | freeze-dried cells^1^ | mustard spinach | no PNSB powder addition | 60.5 | 38.9 | - | - | 48.9 | Kondo *et al.* (2004) |
|  | freeze-dried cells^2^ |  |  | 4.5 | -10.0 | - | - | -1.3 |  |
|  | freeze-dried cells^3^ |  |  | -1.6 | 3.4 | - | - | 25.6 |  |
| *Rhodopseudomonas* sp. BL6 | 2.24 ∙ 10^11^ autoclaved cells^4^ | tomato | sterile water | - | - | - | 1.7 | - | Lee *et al.* (2008) |
| *Rhodopseudomonas* sp. KL9 | 2.24 ∙ 10^11^ autoclaved cells^4^ |  |  |  |  |  | -31.7 |  |  |
| *Rhodobacter sphaeroides* NR3 | 0.4 g dried cells | spinach | no PNSB powder addition | -30.8 (56.3) | -36.8 (71.4) | - | - | 31.3 (14.2) | Kondo *et al.* (2008) |
|  | 0.8 g dried cells |  |  | -12.8 (46.9) | -36.1 (42.9) |  |  | 138.2 (21.8) |  |
|  | 1.6 g dried cells |  |  | 5.1 (28.1) | -15.8 (28.6) |  |  | 77.9 (16.7) |  |
|  | 0.28 g dried cells | mustard spinach |  | 28.9 | 13.6 | - | - | 20.9 |  |
|  | 0.56 g dried cells |  |  | 50.0 | 9.1 |  |  | 6.6 |  |
|  | 1.12 g dried cells |  |  | 42.1 | 9.1 |  |  | 4.1 |  |
| *Rhodobacter sphaeroides* NR3 | 1.25 g dried cells^5^ | tomato | 1.25 g (NH_4_)_2_SO_4_ | - | - | -3.3 | -15.6 | - | Kondo *et al.* (2010) |
|  | 1.25 g dried cells^6^ |  |  |  |  | -3.2 | -18.0 |  |  |
|  | 2.5 g dried cells^5^ |  | 2.5 g (NH_4_)_2_SO_4_ |  |  | 3.3 | 18 |  |  |
|  | 2.5 g dried cells^6^ |  |  |  |  | -5.3 | -1.3 |  |  |

N.A. = not available; numbers in parenthesis indicate experiments with sterilized soil

^1^irrigation (daily irrigation of 0.1 g/L PNSB cells dissolved in 10% Hoagland solution; quantity not specified): treatment with 100% blue light (470 nm); ^2^irrigation (daily irrigation of 0.1 g/L PNSB cells dissolved in 10% Hoagland solution; quantity not specified): treatment with 100% red light (660 nm);^3^irrigation (daily irrigation of 0.1 g/L PNSB cells dissolved in 10% Hoagland solution; quantity not specified): treatment with 20% blue light (470 nm) - 80% red light (660 nm);

^4^4.0 ∙ 10^9^ cells per day for 8 weeks (400 mL bacterial suspension at a concentration of 10^7^ cells mL^-1^)

^5^dosed once; ^6^split application over 10 times

## 4.2 Effect of living cells

Table 6: Effect of living PNSB supplementation on plant performance. Relative change in characteristics compared to the control. Numbers are presented in percentages (%).

| PNSB strain | Dosage | Type of plant | Control | Effect (% of control) | | | | | | | Reference |
| --- | --- | --- | --- | --- | --- | --- | --- | --- | --- | --- | --- |
|  |  |  |  | Seed germination rate | Shoot fresh weight | Shoot dry weight | Shoot length | Root dry weight | Root length | Total Chlorophyll content |  |
| *Rhodobacter capsulatus* DSM 155 | N.A.^1^ | rice | uninoculated seeds^1^ | - | - | 23.6 | 1.9 | - | - | - | Elbadry *et al.* (1999) |
| *Rhodobacter capsulatus* DSM 155 | 1.32 ∙ 10^10^ cells^2^ | rice^4^ | nitrogen-free nutrient solution | - | - | 70.7 | 60.5 | 1.0 | -23.2 | - | Elbadry and Elbanna (1999) |
|  | 1.32 ∙ 10^10^ cells^3^ |  | nutrient solution |  |  | -1.4 | 6.2 | 1.3 | -23.0 |  |  |
| *Rhodopseudomonas palustris* KN122 | 2.26 ∙ 10^11^ cells^5^ | rice | no inoculation | - | - | 0.9 | - | - | - | - | Harada *et al.* (2005) |
|  | 2.26 ∙ 10^11^ cells^6^ |  | rice straw |  |  | 0.4 |  |  |  |  |  |
| *Rhodopseudomonas* sp. BL6 | 5 ∙ 10^7^ cells^7^ | tomato | sterile water | 7.6 | - | 174.4^8^ | 36.5 | - | - | - | Koh and Song (2007) |
| *Rhodopseudomonas* sp. KL9 | 5 ∙ 10^7^ cells^7^ |  |  | 31.9 |  | 263.8^8^ | 70.8 |  |  |  |  |
| *Rhodopseudomonas* sp. BL6 | 2.24 ∙ 10^11^ cells^9^ | tomato | sterile water | - | - | 46.7 | 17.6 | 11.9 | - | - | Lee *et al.* (2008) |
| *Rhodopseudomonas* sp. KL9 | 2.24 ∙ 10^11^ cells^9^ |  |  |  |  | 120.6 | 34.6 | 78.6 |  |  |  |
| *Rhodobacter capsulatus* DSM 155 | N.A.^1^ | rice^10^ | no fertilizer | - | 19.0 | - | 6.9 | - | - | - | Gamal-Eldin and Elbanna (2011) |
| *Rhodopseudomonas palustris* | 4.0 ∙ 10^10^ cells^11^ | Chinese dwarf cherry | culture suspension without cells | - | 44.1 | - | - | - | - | 4.8 | Yin *et al.* (2012) |
| *Rhodopseudomonas* sp. (ISP-1) | 2.4 ∙ 10^12^ cells^12^ | stevia | sterile water | - | 26.3 | - | - | - | - | 48.7 | Wu *et al.* (2013) |
|  | 3.0 ∙ 10^12^ cells^13^ |  |  |  | 31.6 |  |  |  |  | 29.5 |  |
|  | 2.7 ∙ 10^12^ cells^14^ |  |  |  | 68.3 |  |  |  |  | 87.2 |  |
| *Rhodopseudomonas palustris* PS3 | 4.8 ∙ 10^9^ CFU^15^ | mustard spinach | 50% standard amount of chemical fertilizer | - | 44.4 (26.9) | 66.7 (46.8) | - | - | - | - | Wong *et al*. (2014) |
| *Rhodopseudomonas palustris* YSC3 | 4.8 ∙ 10^9^ CFU^15^ |  |  |  | -3.7 (4.6) | 15.4 (29.9) |  |  |  |  |  |
| *Rhodopseudomonas palustris* YSC4 | 4.8 ∙ 10^9^ CFU^15^ |  |  |  | 14.8 (11.1) | 35.9 (23.4) |  |  |  |  |  |
| *Rhodopseudomonas palustris* TK103 | 10^8^ cells | rice | uninoculated carrier | - | - | 67.7 | 22.9 | 78.8 | 10.6 | - | Kantha *et al.* (2015) |
|  | 10^8^ cells^16^ |  |  |  |  | 73.1 | 38.1 | 210.0 | 98.0 |  |  |
| *Rhodopseudomonas palustris* PP803 | 10^8^ cells |  |  |  |  | 77.4 | 29.2 | 154.5 | 8.3 |  |  |
|  | 10^8^ cells^16^ |  |  |  |  | 115.4 | 45.2 | 250.0 | 105 |  |  |
| *Rhodopseudomonas palustris* P1 | 10^8^ cells |  |  |  |  | 48.4 | 18.8 | 72.7 | 11.1 |  |  |
|  | 10^8^ cells^16^ |  |  |  |  | 94.2 | 35.7 | 210.1 | 80.0 |  |  |
| *Rhodopseudomonas palustris* TN114 | 9 ∙ 10^11^ cells^17^ | rice | uninoculated carrier | - | - | - | -1.1 | - | - | - | Kantachote *et al.* (2016) |
| *Rhodopseudomonas palustris* PP803 | 9 ∙ 10^11^ cells^17^ |  |  |  |  |  | -0.4 |  |  |  |  |
| *Rhodopseudomonas palustris* TK103 | 9 ∙ 10^11^ cells^17^ |  |  |  |  |  | -0.5 |  |  |  |  |
| *Rhodopseudomonas palustris* TN114 | 7.5 ∙ 10^11^ cells^18^ |  |  |  |  |  | 6.1 |  |  |  |  |
| *Rhodopseudomonas palustris* PP803 | 7.5 ∙ 10^11^ cells^18^ |  |  |  |  |  | 9.9 |  |  |  |  |
| *Rhodopseudomonas palustris* TK103 | 7.5 ∙ 10^11^ cells^18^ |  |  |  |  |  | 4.1 |  |  |  |  |
| *Rhodopseudomonas palustris* CS2 | N.A.^1^ | mungo beans | uninoculated seeds | - | 21.4 | 50.0^10^ | 17.0 | - | 21.7 | - | Batool *et al.* (2017) |
| *Rhodopseudomonas faecalis* SS5 | N.A.^1^ |  |  |  | 14.3 | 0.0 | 12.8 |  | 18.8 |  |  |
| *Rhodopseudomonas palustris* CS2 and  *Rhodopseudomonas faecalis* SS5 | N.A.^1^ |  |  |  | - | - | 15.4 |  | 24.2 |  |  |
| *Rhodopseudomonas palustris* GJ-22 | N.A.^19^ | tobacco | ultrapure water | 20.5 | 30.8 [33.7]^20^ | - | 55.4 | - | 49.5 | - | Su *et al.* (2017) |
| *Rhodopseudomonas palustris* | 5 ∙ 10^10^ cells | tobacco | same amount of autoclaved cells | - | - | 6.8 | 14.1 | 31.5 | - | - | Hua *et al.* (2014) |
| *Rhodopseudomonas palustris* | N.A.^21^ | pak choi | sterile water | - | 13.1^22^ | - | - | - | - | - | Xu *et al.* (2016) |
|  |  |  |  |  | 36.5^23^ |  |  |  |  |  |  |
|  |  |  |  |  | 78.9^24^ |  |  |  |  |  |  |
| *Rhodopseudomonas palustris* PS3 | 3.5 ∙ 10^10^ CFU^22^ | pak choi | Hoaglang solution | - | -22.4 | -21.7 | - | - | - | - | Hsu *et al.* (2015) |
|  |  | lettuce |  |  | 23.4 | 7.5 |  |  |  |  |  |
| *Rhodopseudomonas palustris* BCRC16408 |  | pak choi |  |  | -47.1 | -45.5 |  |  |  |  |  |
|  |  | lettuce |  |  | -7.8 | -10.5 |  |  |  |  |  |
| *Rhodopseudomonas palustris* | 10^9^ cells | stevia | water | - | - | 4.3^23^ | - | - | - | - | Xu *et al.* (2018) |
|  |  |  |  |  |  | 2.5^24^ |  |  |  |  |  |
| *Rhodopseudomonas palustris* C1 | 2.5 ∙ 10^11^ cells | rice^24^ | rice nutrient solution | - | - | 44.8 | 7.0 | 69.0 | - | 1.2 | Nookongbut *et al.* (2018) |
|  |  |  | rice nutrient solution containing As(III) |  |  | 42.5 | 36.0 | 62.4 |  | 61.2 |  |
|  |  |  | rice nutrient solution containing As(IV) |  |  | 122.7 | 8.3 | 70.0 |  | 22.6 |  |
| *Rubrivivax benzoatilyticus* C31 |  |  | rice nutrient solution |  |  | 2.6 | -2.8 | 25.2 |  | -3.2 |  |
|  |  |  | rice nutrient solution containing As(III) |  |  | 40.3 | 5.7 | 20.0 |  | 44.4 |  |
|  |  |  | rice nutrient solution containing As(IV) |  |  | 110.7 | 8.8 | 42.5 |  | 11.8 |  |
| *Rhodopseudomonas palustris* C1 and  *Rubrivivax benzoatilyticus* C31 |  |  | rice nutrient solution |  |  | 94.3 | 13.4 | 81.4 |  | 8.4 |  |
|  |  |  | rice nutrient solution containing As(III) |  |  | 58.0 | 34.6 | 32.5 |  | 74.9 |  |
|  |  |  | rice nutrient solution containing As(IV) |  |  | 249.1 | 32.3 | 121.7 |  | 30.5 |  |

Numbers in parenthesis indicate experiments with new (not stored) seeds

^1^Concerns seed coating

^2^In hydroponic solution, without N supplementation

^3^In hydroponic solution, with N supplementation

^4^Four rice varieties, mean values are presented

^5^Rice straw not applied – triple inoculation

^6^Rice straw applied – triple inoculation

^7^Seeds inoculated with 5 mL of alive or autoclaved bacterial suspension (10^7^ cells mL^-1^)

^8^Concerns whole seedling

^9^4.0 ∙ 10^9^ cells per day (400 mL bacterial suspension at a concentration of 10^7^ cells mL^-1^)

^10^Average values from two fields

^11^2.0∙ 10^9^ cells per week (10 mL bacterial suspension at a concentration of 2.0∙10^9^ MPN mL^-1^)

^12^foliar spray: leaves sprayed with 50 mL suspension of 0.6·10^10^ cells/mL once a day for 8 days; ^13^rhizosphere irrigation: soil was irrigated with 100 mL suspension of 3·10^10^ cells/mL once; ^14^spray + irrigation: leaves sprayed with 50mL suspension of 0.3·10^10^ cells/mL once a day for 8 days and soil was irrigated with 100mL suspension of 1.5·10^10^ cells/mL once

^15^50% of standard amount of chemical fertilizer + *R. palustris* strain (8 mL of suspension (1.5·10^8^ CFU mL^-1^) applied once per week for 4 weeks)

^16^under salt stress (0.25% NaCl)

^17^experiments performed in organic fied

^18^experiments performed in saline field

^19^foliar spray to soaking wet of suspension with density of 6∙10^7^ CFU mL^-1^

^20^Both values concern yield increase (kg/ha), number in bracket indicates first class tobacco yield increase

^21^1-5 mL foliar spray of suspension with density of 10^10^ CFU mL^-1^; ^22^PNSB grown in medium supplemented with tryptophan; ^23^PNSB grown in stevia residue extract supplemented with tryptophan; ^24^PNSB grown in stevia residue extract supplemented with NH_4_Cl

^22^50% Hoaglang solution with PNSB suspension of 10^6^ CFU mL^-1^, in 35 L tank

^23^dry weight of leaves in unfertilized field; ^24^dry weight of leaves in fertilized field

^24^values presented are the average of two varieties of rice plant

Table 7: Effect of PNSB (living cells) supplementation on edible plant biomass. Relative change in qualitative and quantitative characteristics compared to the control. Numbers are presented in percentages (%)

| PNSB strain | Dosage | Type of crop/ leafy vegetable | Control | % of control | | | | | | | | Reference |
| --- | --- | --- | --- | --- | --- | --- | --- | --- | --- | --- | --- | --- |
|  |  |  |  | Fruit per flower^1^ | Total number of crops | Average fresh weight per crop^2^ | Total weight per harvest (yield) | Total water content | Total sugar content | Brix sugar content | Protein content |  |
| N.A. | N.A. | mandarin | 1L of 1:1000 diluted commercial liquid fertilizer (N: 10%; P: 5%; K: 5%) every 10 days | - | 9.1 | 16.7 | 27.3 | - | 5.8 | - | - | Kobayashi and Tchan (1973) |
| *Rhodopseudomonas capsulata* | N.A. | rice | inorganic fertilizer | - | - | - | 20.4 | - | - | - | - | Yoshida *et al.* (1991) |
| N.A. | N.A. | persimmon | inorganic fertilizer | - | 34.4 | -14 | 15.5 | -1.7 | 15.0 | 11.6 | - | Kobayashi and Kobayashi (1995) |
| Mixed culture of Rhodospirillaceae | N.A. | grape | water | 1.9 | - | 2.5 | 6.9 | - | - | - | - | Shi *et al.* (1995) |
| *Rhodopseudomonas palustris* | 8.25 ∙ 10^12^ MPN^3^ | mushroom^4^ | water | - | 7.4 | 2.5 | 10.1 | 9.8 | - | - | 2.2 | Han (1999) |
|  | 9.90 ∙ 10^12^ MPN^3^ |  |  |  | 18.9 | -0.2 | 14.1 | 7.3 |  |  | 0.6 |  |
|  | 12.4 ∙ 10^12^ MPN^3^ |  |  |  | 20.6 | -2.0 | 18.1 | 5.4 |  |  | -1.7 |  |
|  | 16.5 ∙ 10^12^ MPN^3^ |  |  |  | 26.3 | -3.3 | 22.1 | 2.3 |  |  | 1.5 |  |
| *Rhodobacter capsulatus* DSM 155^5^ | N.A. | rice | uninoculated seeds | 3.9 | - | -9.1 | 30.2 | - | - | - | 7.1^6^ | Elbadry *et al.* (1999) |
|  |  |  |  | - |  | - | 32.8 |  |  |  | - |  |
|  |  |  |  |  |  |  | 18.7 |  |  |  |  |  |
|  |  |  |  |  |  |  | 19.8 |  |  |  |  |  |
| *Rhodopseudomonas palustris* KN122 | 2.26 ∙ 10^11^ cells^7^ | rice | no inoculation | - | - | 0.4 | 8.9 | - | - | - | - | Harada *et al.* (2005) |
|  | 1.7 ∙ 10^11^ cells^8^ |  | rice straw |  |  | -0.8 | 16.4 |  |  |  |  |  |
|  | 2.26 ∙ 10^11^ cells^9^ |  | rice straw |  |  | -0.8 | 24.1 |  |  |  |  |  |
| *Rhodopseudomonas* sp. BL6 | 2.24 ∙ 10^11^ cells^10^ | tomato | sterile water | 35.9 | - | - | 20.9 | - | - | - | - | Lee *et al.* (2008) |
| *Rhodopseudomonas* sp. KL9 | 2.24 ∙ 10^11^ cells^10^ |  |  | 88.6 |  |  | 98.3 |  |  |  |  |  |
| *Rhodobacter capsulatus* DSM 155^5^ | N.A.^11^ | rice | no fertilizer | - | - | - | 30.2^17^ | - | - | - | - | Gamal-Eldin and Elbanna (2011) |
|  | N.A.^12^ |  | 50% the recommended chemical N fertilizer rate |  |  |  | 19.4^17^ |  |  |  |  |  |
|  | N.A.^13^ |  | 100% the recommended chemical N fertilizer rate |  |  |  | 14.4^17^ |  |  |  |  |  |
|  | N.A.^14^ |  | manure containing cow dung and rice straw |  |  |  | 23.1^17^ |  |  |  |  |  |
|  | N.A.^15^ |  | 50% the recommended chemical N fertilizer rate and manure containing cow dung and rice straw |  |  |  | 15.7^17^ |  |  |  |  |  |
|  | N.A.^16^ |  | 100% the recommended chemical N fertilizer rate and manure containing cow dung and rice straw |  |  |  | 14.3^17^ |  |  |  |  |  |
| *Rhodopseudomonas* sp. (ISP-1) | 2.4 ∙ 10^12^ cells^18^ | stevia leaves^21^ | sterile water | - | - | - | - | - | 77.1^22^ | - | - | Wu *et al.* (2013) |
|  | 3.0 ∙ 10^12^ cells^19^ |  |  |  |  |  |  |  | 57.6^22^ |  |  |  |
|  | 2.7 ∙ 10^12^ cells^20^ |  |  |  |  |  |  |  | 115.9^22^ |  |  |  |
| *Rhodopseudomonas palustris* TN114 | 9 ∙ 10^11^cells^23^ | rice | uninoculated carrier | 20.6 | - | - | 19.4 | - | - | - | - | Kantachote *et al.* (2016) |
| *Rhodopseudomonas palustris* PP803 | 9 ∙ 10^11^ cells^23^ |  |  | 21.8 |  |  | 5.8 |  |  |  |  |  |
| *Rhodopseudomonas palustris* TK103 | 9 ∙ 10^11^ cells^23^ |  |  | 19.1 |  |  | 6.8 |  |  |  |  |  |
| *Rhodopseudomonas palustris* TN114 | 7.5 ∙ 10^11^ cells^24^ |  |  | 8.4 |  |  | 5.2 |  |  |  |  |  |
| *Rhodopseudomonas palustris* PP803 | 7.5 ∙ 10^11^ cells^24^ |  |  | 17.7 |  |  | 9.0 |  |  |  |  |  |
| *Rhodopseudomonas palustris* TK103 | 7.5 ∙ 10^11^ cells^24^ |  |  | 16.5 |  |  | 6.3 |  |  |  |  |  |
| *Rhodopseudomonas palustris* | 5 ∙ 10^10^ cells | tobacco | same amount of autoclaved cells | - | 19.5^25^ |  |  |  | - | - | - | Hua *et al.* (2014) |
| *Rhodopseudomonas palustris* | N.A.^26^ | pak choi | sterile water | - | - | - | 13.1^27^ | - | - | - | - | Xu *et al.* (2016) |
|  |  |  |  |  |  |  | 36.5^28^ |  |  |  |  |  |
|  |  |  |  |  |  |  | 78.9^29^ |  |  |  |  |  |
| *Rhodopseudomonas palustris* PS3 | 3.5 ∙ 10^10^ CFU^27^ | pak choi | Hoaglang solution | - | - | -22.4 | - | - | - | - | - | Hsu *et al.* (2015) |
|  |  | lettuce |  |  |  | 23.4 |  |  |  |  |  |  |
| *Rhodopseudomonas palustris* BCRC16408 |  | pak choi |  |  |  | -47.1 |  |  |  |  |  |  |
|  |  | lettuce |  |  |  | -7.8 |  |  |  |  |  |  |
| *Rhodopseudomonas palustris* | 10^9^ cells | stevia | water | - | - | - | 4.3^28^ | - | - | - | - | Xu *et al.* (2018) |
|  |  |  |  |  |  |  | 2.5^29^ |  |  |  |  |  |

N.A. = not available; N/A = not applicable; MPN = most probable number of living cells;

^1^in the case of rice: grain/panicle

^2^in the case of rice: weight of 1000 grains

^3^dosed in four applications;

^4^mushroom is not a crop (fungus) but was included here since it is cultivated as a plant

^5^concerns lysimeter experiment

^6^in this case N content

^7^rice straw not applied – triple inoculation; ^7^rice straw applied – single inoculation; ^7^rice straw applied – triple inoculation

^10^4.0 ∙ 10^9^ cells per day for 8 weeks (400 mL bacterial suspension at a concentration of 10^7^ cells mL^-1^)

^11^no fertilizer addition (organic or inorganic) + seedling inoculation; ^12^50% the recommended chemical N fertilizer rate + seedling inoculation; ^13^100% the recommended chemical N fertilizer rate + seedling inoculation; ^14^manure containing cow dung and rice straw + seedling inoculation; ^15^50% the recommended chemical N fertilizer rate and manure containing cow dung and rice straw + seedling inoculation; ^16^100% the recommended chemical N fertilizer rate and manure containing cow dung and rice straw + seedling inoculation; ^17^average values from 2 fields

^18^foliar spray: leaves sprayed with 50 mL suspension of 0.3·10^10^ cells/mL once a day for 8 days; ^19^rhizosphere irrigation: soil was irrigated with 100 mL suspension of 3·10^10^ cells/mL once; ^20^spray + irrigation: leaves sprayed with 50mL suspension of 0.3·10^10^ cells/mL once a day for 8 days and soil was irrigated with 100mL suspension of 1.5·10^10^ cells/mL once; ^21^concerns the old leaves (not newly formed); ^22^soluble sugar

^23^experiments conducted in organic paddy field

^24^experiments conducted in saline paddy field

^25^number of leaves

^26^1-5 mL foliar spray of suspension with density of 10^10^ CFU mL^-1^; ^27^PNSB grown in medium supplemented with tryptophan; ^28^PNSB grown in stevia residue extract supplemented with tryptophan; ^29^PNSB grown in stevia residue extract supplemented with NH_4_Cl

^27^50% Hoaglang solution with PNSB suspension of 10^6^ CFU mL^-1^, in 35 L tank

^28^dry weight of leaves in unfertilized field; ^29^dry weight of leaves in fertilized field

Table 8: Effect of living PNSB supplementation on the content of pigments: chlorophyll a and b, individual carotenoid pigment (β-carotene, lycopene, cryptoxanthin and zeaxanthin) and total carotenoid content content of crops and leafy vegetables, in comparison to the control. Numbers are presented in percentages (%).

| PNSB strain | Dosage | Type of crop/ leafy vegetable | Control | Chlorophyll a | Chlorophyll b | β-carotene | Lycopene | Cryptoxanthin | Zeaxanthin | Total carotenoid | Reference |
| --- | --- | --- | --- | --- | --- | --- | --- | --- | --- | --- | --- |
| N.A. | N.A. | persimmon | inorganic fertilizer | - | - | -5.8 | 52.8 | 20.3 | 16.8 | 19.6 | Kobayashi and Kobayashi (1995) |
| *Rhodopseudomonas* sp. BL6 | 2.24 ∙ 10^11^ cells^1^ | tomato | sterile water | - | - | - | 1.7 | - | - | - | Lee *et al.* (2008) |
|  | 2.24 ∙ 10^11^ cells^1^ |  |  |  |  |  | 48.3 |  |  |  |  |
| *Rhodopseudomonas* sp. (ISP-1) | 2.4 ∙ 10^12^ cells^2^ | stevia leaves^5^ | sterile water | 50.0 | 46.5 | - | - | - | - | - | Wu *et al.* (2013) |
|  | 3.0 ∙ 10^12^ cells^3^ |  |  | 29.7 | 29.1 |  |  |  |  |  |  |
|  | 2.7 ∙ 10^12^ cells^4^ |  |  | 90.5 | 81.4 |  |  |  |  |  |  |
| *Rhodopseudomonas palustris* C1 | 2.5 ∙ 10^11^ cells | rice^6^ | rice nutrient solution | 2.8 | -2.0 | - | - | - | - | -1.1 | Nookongbut *et al.* (2018) |
|  |  |  | rice nutrient solution containing As(III) | 61.7 | 60.7 | - | - | - | - | -17.8 |  |
|  |  |  | rice nutrient solution containing As(IV) | 18.6 | 32.0 | - | - | - | - | -5.9 |  |
| *Rubrivivax benzoatilyticus* C31 |  |  | rice nutrient solution | -2.2 | -4.7 | - | - | - | - | 3.8 |  |
|  |  |  | rice nutrient solution containing As(III) | 46.7 | 40.4 | - | - | - | - | -12.2 |  |
|  |  |  | rice nutrient solution containing As(IV) | 7.8 | 20.7 | - | - | - | - | -3.6 |  |
| *Rhodopseudomonas palustris* C1 and  *Rubrivivax benzoatilyticus* C31 |  |  | rice nutrient solution | 7.6 | 10.8 | - | - | - | - | -3.9 |  |
|  |  |  | rice nutrient solution containing As(III) | 73.6 | 77.4 | - | - | - | - | -25.7 |  |
|  |  |  | rice nutrient solution containing As(IV) | 31.4 | 29.5 | - | - | - | - | -10.2 |  |

N.A. = not available

^1^4.0 ∙ 10^9^ cells per day for 8 weeks (400 mL bacterial suspension at a concentration of 10^7^ cells mL^-1^)

^2^foliar spray: leaves sprayed with 50 mL suspension of 0.3·10^10^ cells/mL once a day for 8 days; ^3^rhizosphere irrigation: soil was irrigated with 100 mL suspension of 3·10^10^ cells/mL once; ^4^spray + irrigation: leaves sprayed with 50mL suspension of 0.3·10^10^ cells/mL once a day for 8 days and soil was irrigated with 100mL suspension of 1.5·10^10^ cells/mL once; ^5^concerns the old leaves (not newly formed)

^6^values presented are the average of two varieties of rice plant

## Effect of PNSB extracted substances

Table 9: Effect of PNSB (extracted substances) supplementation on plant performance. Relative change in characteristics compared to the control (distilled water). Numbers are presented in percentages (%).

| PNSB strain | Dosage  (μΜ ALA) | Type of plant | Control | Effect (% of control) | | | | Reference |
| --- | --- | --- | --- | --- | --- | --- | --- | --- |
|  |  |  |  | Plant fresh weight | Plant dry weight | Root length | Total Chlorophyll content |  |
| *Rhodopseudomonas palustris* TN114 | 2.67 | rice | distilled water | 17.7 | 78.3 | 157.9 | 13.3 | Nunkaew *et al.* (2014) |
| *Rhodopseudomonas palustris* PP803 | 2.11 |  |  | -7.4 | 1.1 | 137.1 | 16.2 |  |

# Economic calculations

## PNSB biomass production costs

In order to calculate the production costs of the dried PNSB, Alloul *et al.* (2019) used a spray dryer with a heat demand of 1.75 kWh_th_ kg_water removed_^-1^. The biomass concentration after centrifugation is estimated as 200 g_VSS_ L^-1^ (Davis *et al.*, 2016). A biomass recovery of 95% after the spray dryer is expected and thus the heat demand becomes:

$$\frac{{1.75 kWh}_{th} {{kg}_{water}}^{-1}}{0.2 kg VSS {{kg}_{water}}^{-1}\cdot0.95}=9.2\frac{{kWh}_{th}}{{kg}_{VSS}}$$

The ratio of 0.75 kg_VSS_ kg_TSS_ ^-1^ given by Alloul *et al.* (2019) was then used to transform this to 6.91 kWh_th_ kg_TSS_^-1^. The average natural gas price in Europe in 2018 was €0.03 kWh_th_^-1^ (Eurostat, 2019) and thus the OPEX for spray drying results in € 0.21 kg_DW_^-1^.

The CAPEX for the spray dryer accounts for €460,000 unit^-1^ with a depreciation of 10 years and an interest rate of 1.67%. To subtract this total investment cost (TIC) from the production cost an annualized investment cost (AIC) has to be determined, which results in an AIC of €503,302 year^-1^.

$$AIC= \frac{TIC}{a_{i,n}}$$

Where:

a_i,n_ = [(1+i)^n^-1]/[i·(1+i)^n^]

i = annual interest rate (1.67%)

n = economic lifetime of the unit in years (10 years)

Furthermore, the annual biomass production can be calculated as:

$$Biomass production \left( {kg}_{DW} {year}^{-1} \right)=\frac{\frac{2,427 {kg}_{COD}}{day}\cdot\frac{0.70 {kg}_{VSS}}{{kg}_{COD}}\cdot\frac{260 working days}{year}}{\frac{0.75 {kg}_{VSS}}{{kg}_{TSS}}}$$

This results in a production of 589 tons_DW_ year^-1^ and therefore the CAPEX of the spray dryer expressed per biomass unit becomes €0.85 kg_DW_^-1^. In total, a production cost of € 1.06 kg_DW_^-1^ can thus be avoided if the viable biomass is used instead of going to a dried PNSB product.

## Price based on nitrogen and phosphorus content

The commercial price for the liquid organic nitrogen fertilizer (DCM, 2019) containing 9% N was obtained through personal communication while the price for the organic phosphorus fertilizer (bone meal) containing 16% P were found online (bol.com, 2019):

$$Liquid organic nitrogen fertilizer: \frac{\frac{€5.6}{{kg}_{product}}}{\frac{0.09 {kg}_{N}}{{kg}_{product}}}= \frac{€61.8}{{kg}_{N}}$$

$$Bone meal: \frac{\frac{€10}{{kg}_{product}}}{\frac{0.16 {kg}_{P}}{{kg}_{product}}}= \frac{€62.5}{{kg}_{P}}$$

The price of dried PNSB biomass was based on the cost estimations made by Alloul *et al.* (2019), increased by 30% to account for packaging, logistics and profit. It should be noted that the N and P content of PNSB originate from own data for dried biomass of *Rhodobacter* sp:

$$PNSB biomass: \frac{\frac{€13}{{kg}_{product}}}{\frac{0.085 {kg}_{N}}{{kg}_{product}}}= \frac{€153}{{kg}_{N}}$$

$$PNSB biomass: \frac{\frac{€13}{{kg}_{product}}}{\frac{0.024 {kg}_{P}}{{kg}_{product}}}= \frac{€542}{{kg}_{P}}$$

## Indole-3-acetic acid (IAA) and 5-aminolevulinic acid (ALA) production from PNSB

The assumptions and calculations for the PNSB-derived ALA and IAA are presented in Table 10. Given that the production prices through conventional processes are not available, the estimations of the IAA and ALA production costs are based on the production price of PNSB biomass. Finally, the prices of commercial IAA and ALA products were used for comparison.

Table 10: Assumptions and calculations of the prices for indole-3-acetic acid (IAA) and 5-aminolevulinic acid (ALA) from purple non-sulphur bacteria.

| **Parameter** | **Value** | | **Unit** | **Reference/Comment** |
| --- | --- | --- | --- | --- |
|  | **Lower level** | **Higher level** |  |  |
| **Estimated prices from PNSB** | | | | |
| Prices | 11.6 | | € kg_DW_^-1^ | Dry – wet biomass, based on SI section 5.1 |
| Biomass concentration | 2 | | kg m^-3^ | Carlozzi and Sacchi (2001) |
| Price per m^3^ | 44.7 | | € m^-3^ | Calculated |
| IAA concentration | 0.65 | 52 | mg_IAA_ L^-1^ | Extracellular levels, based on manuscript section 2.2.1 |
|  | 0.00065 | 0.052 | kg_IAA_ m^-3^ |  |
| Price IAA from PNSB | 0.0085 | 13.6 | € m^-3^ | Calculated |
|  | 35,692 | 446 | € kg_IAA_^-1^ |  |
| ALA concentration | 0.23 | 7.9 | mg_ALA_ L^-1^ | Extracellular levels, based on manuscript section 2.2.1 |
|  | 0.00023 | 0.0079 | kg_ALA_ m^-3^ |  |
| Price of ALA from PNSB | 0.4002 | 68.73 | € m^-3^ | Calculated |
|  | 100,870 | 2,937 | € kg_ALA_^-1^ |  |
| **Commercial prices** | | | | |
| Commercial price of ALA | 2,000 | 10,000 | USD kg^-1^ | Alibaba (2019a) |
|  | 1,740 | 8,700 | € kg^-1^ | 1 USD = €0.87 |
| Commercial price of IAA | 15 | 300 | USD kg^-1^ | Alibaba (2019b) |
|  | 13.05 | 261 | € kg^-1^ | 1 USD = €0.87 |
